# CRNDE mediated hnRNPA2B1 stability facilitates nuclear export and translation of KRAS in colorectal cancer

**DOI:** 10.1038/s41419-023-06137-9

**Published:** 2023-09-16

**Authors:** Ya Lu, Renrui Zou, Quan Gu, Xinyue Wang, Junying Zhang, Rong Ma, Ting Wang, Jianzhong Wu, Jifeng Feng, Yuan Zhang

**Affiliations:** 1 Jiangsu Cancer Hospital & Jiangsu Institute of Cancer Research & The Affiliated Cancer Hospital of Nanjing Medical University, Nanjing, China; 2 Research Center of Clinical Oncology, Jiangsu Cancer Hospital & Jiangsu Institute of Cancer Research & The Affiliated Cancer Hospital of Nanjing Medical University, Nanjing, China; 3 Department of Cell Biology, School of Basic Medical Sciences, Nanjing Medical University, Nanjing, China

**Keywords:** Colon cancer, Prognostic markers

## Abstract

Development of colorectal cancer (CRC) involves activation of Kirsten rat sarcoma viral oncogene homolog (KRAS) signaling. However, the post-transcriptional regulation of KRAS has yet to be fully characterized. Here, we found that the colorectal neoplasia differentially expressed (CRNDE)/heterogeneous nuclear ribonucleoprotein A2/B1 (hnRNPA2B1) axis was notably elevated in CRC and was strongly associated with poor prognosis of patients, while also significantly promoting CRC cell proliferation and metastasis both in vitro and in vivo. Furthermore, CRNDE maintained the stability of hnRNPA2B1 protein by inhibiting E3 ubiquitin ligase TRIM21 mediated K63 ubiquitination-dependent protein degradation. CRNDE/hnRNPA2B1 axis facilitated the nuclear export and translation of KRAS mRNA, which specifically activated the MAPK signaling pathway, eventually accelerating the malignant progression of CRC. Our findings provided insight into the regulatory network for stable hnRNPA2B1 protein expression, and the molecular mechanisms by which the CRNDE/hnRNPA2B1 axis mediated KRAS nucleocytoplasmic transport and translation, deeply underscoring the bright future of hnRNPA2B1 as a promising biomarker and therapeutic target for CRC.

**Figure:**
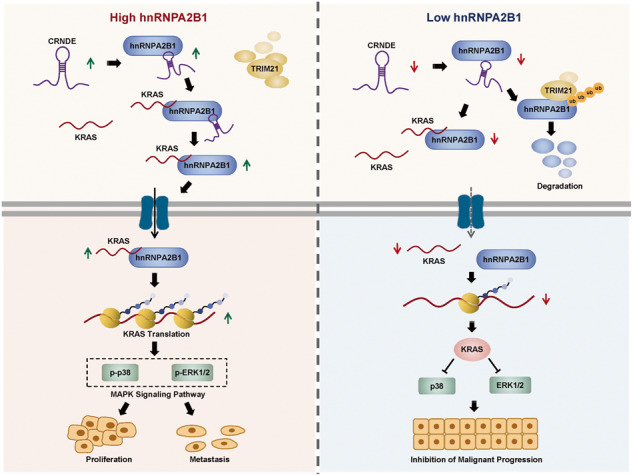
By hindering hnRNPA2B1 from binding to the E3 ubiquitin ligase TRIM21, whose mediated ubiquitin-dependent degradation was thereby inhibited, CRNDE protected the stability of hnRNPA2B1’s high protein expression in CRC. Supported by the high level of the oncogenic molecule CRNDE, hnRNPA2B1 bound to KRAS mRNA and promoted KRAS mRNA nucleus export to enter the ribosomal translation program, subsequently activating the MAPK signaling pathway and ultimately accelerating the malignant progression of CRC.

By hindering hnRNPA2B1 from binding to the E3 ubiquitin ligase TRIM21, whose mediated ubiquitin-dependent degradation was thereby inhibited, CRNDE protected the stability of hnRNPA2B1’s high protein expression in CRC. Supported by the high level of the oncogenic molecule CRNDE, hnRNPA2B1 bound to KRAS mRNA and promoted KRAS mRNA nucleus export to enter the ribosomal translation program, subsequently activating the MAPK signaling pathway and ultimately accelerating the malignant progression of CRC.

## Introduction

Colorectal cancer (CRC) is one of the most common solid malignancies and the second leading cause of cancer-related mortality worldwide [1]. Due to the delay in diagnosis and the lack of effective therapeutic targets [2], chemotherapy and targeted therapies have not fundamentally led to the desired 5-year survival outcomes for CRC patients [3]. Therefore, it is imperative to further study the molecular mechanisms involved in the development of CRC to find more reliable therapeutic strategies for CRC.

Heterogeneous nuclear ribonucleoproteins (hnRNPs), a principal class of classical RNA-binding proteins (RBPs), occupy a considerable proportion in eukaryotic cells and serve as the key participants mediating the entire cycle of nucleic acid metabolism [4, 5]. The long non-coding RNAs (lncRNAs)/hnRNPs interaction network has been known to play significant roles in diverse biological processes involved in cancer initiation and development [6, 7]. Recent emerging insights into the interplay between lncRNAs and RBPs in cancer have also led to a clear understanding of the value of lncRNA/RBP in exploring novel ideas for cancer therapy [8, 9]. As score member of hnRNPs, heterogeneous ribonucleoprotein A2/B1 (hnRNPA2B1) has been reported to be involved in RNA transcription, splicing, trafficking, stability and translation as lncRNA/hnRNPA2B1 [10]. For example, Linc01232/hnRNPA2B1 promotes pancreatic cancer metastasis via regulating the alternative splicing of A-Raf [11], miR503HG/hnRNPA2B1 inhibits hepatocellular carcinoma progression by reducing the stability of p52 and p65 mRNA [12], and NEAT1/hnRNPA2B1 facilitates fatty acid metabolism through increasing RPRD1B mRNA stability in gastric cancer [13]. Meanwhile, Some studies have noted the biological contribution of lncRNA/hnRNPA2B1 to CRC progression, such as MIR100HG/hnRNPA2B1 [14], H19/hnRNPA2B1 [15] and RP11/hnRNPA2B1 [16]. However, the role of colorectal neoplasia differentially expressed (CRNDE)/hnRNPA2B1 axis in CRC and how CRNDE modulates hnRNPA2B1 are less well understood.

Kirsten rat sarcoma viral oncogene homolog (KRAS) is a well-known small GTPases protein. Approximately 40% of CRC patients harbor activating missense mutations in KRAS, and they tend to have a poorer prognosis [17, 18]. Unfortunately, KRAS has been deemed a challenging therapeutic target and was once even considered “undruggable” [19], so the treatment of KRAS-mutant CRC patients remains fraught with difficulties. Additionally, it is worth mentioning that aberrant activation of KRAS is responsible for dysregulation of the RAS/MAPK pathway, which is a pivotal and classical cascade response and contributes to the malignant biological behavior of cancer cells [20]. CRNDE and hnRNPA2B1 have been reported as the two activators of MAPK signaling pathway in cancer progression [11, 21, 22]. Nevertheless, how MAPK signaling pathway is regulated by CRNDE/hnRNPA2B1 axis remains to be elucidated.

In this paper, we elucidated the critical oncogenic roles of the CRNDE/hnRNPA2B1 axis in CRC. CRNDE protected hnRNPA2B1 from TRIM21-mediated K63 ubiquitin-dependent degradation. Subsequently, CRNDE cooperated with hnRNPA2B1 to induce nuclear export and translation of KRAS mRNA, which then activated MAPK signaling cascade. Our study provides a strong support for CRNDE/hnRNPA2B1 as a tumor-driver in CRC and a promising therapeutic target for CRC treatment in the future.

## Materials and methods

### Patient specimen

Human CRC tissues and corresponding adjacent noncancerous tissues were collected from the surgical resection of CRC patients at the Affiliated Cancer Hospital of Nanjing Medical University. The specimens were quickly frozen and stored in liquid nitrogen. The diagnosis of CRC was confirmed based on clinical manifestation and pathological examination. None of the patients had received neoadjuvant therapy.

### Immunohistochemistry (IHC) on tissue microarray (TMA)

TMA was constructed as previously described [23, 24]. The TMA containing 87 CRC and 81 normal colorectal tissue samples were obtained from Outdo Biotech Co., Ltd. (HColA180Su13, Shanghai, China). Immunohistochemistry analysis was performed to detect hnRNPA2B1 expression using anti-hnRNPA2B1 (sc-374053; 1:500; Santa Cruz).

### RNA immunoprecipitation (RIP) assay

RIP assays were carried out with Magna RIP RNA-Binding Protein Immunoprecipitation Kit (Millipore, MA, USA) following the manufacturer’s manual. Retrieved RNAs were purified and detected by qPCR to confirm the signals. The relative levels of immunoprecipitated RNAs were normalized to the corresponding input RNA level.

### SUnSET assay

To study the effect of hnRNPA2B1 on mRNA translation, a nonradioactive method based on puromycin, called the SUnSET assay, was applied according to the protocols reported previously [25]. In detail, the cells that to be tested were incubated with puromycin at a final concentration of 10 μg/mL for 10–30 min, after which the total protein lysates were extracted and aligned, and subsequently western blot was performed using anti-puromycin monoclonal antibody to measure relative changes in protein synthesis.

### Statistical analysis

The quantitative data independently collected in triplicate were presented as mean ± standard deviation (SD). Appropriate statistical methods were applied with GraphPad Prism 7.0 software (GraphPad Software, Inc.). The Student’s *t*-test and one-way analysis of variance were used to calculate differences between groups, chi-square test was used to evaluate the relationship between hnRNPA2B1 and the clinicopathological characteristics in CRC cases, and log-rank test was used for survival analysis. Values of *P* < 0.05 were considered statistically significant.

## Results

### hnRNPA2B1 as an oncogene is closely associated with poor outcomes in CRC

**Fig. 1:**
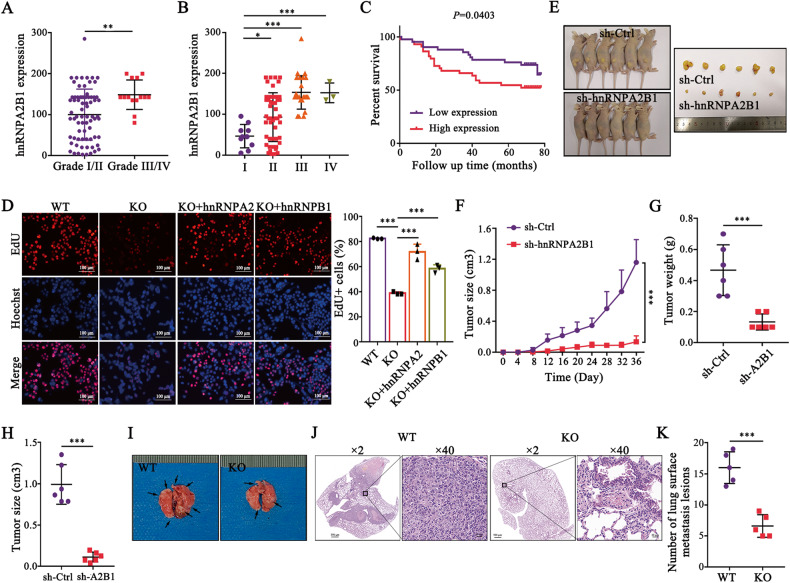
Upregulation of hnRNPA2B1 is associated with CRC poor outcomes. Correlation analysis of hnRNPA2B1 expression with pathological grade (**A**) and clinical TNM stage (**B**) in TMA cohort (*n* = 87). **C** Kaplan–Meier survival curves of CRC patients on TMA grouped by hnRNPA2B1 expression (*P* = 0.0403). **D** EdU assays examined the difference in the role of hnRNPA2 and hnRNPB1 variants on the proliferative capacity of CRC cells. *n* = 3 independent biological replicates. **E** Representative images of mice-bearing tumors derived from xenograft nude mouse model injected subcutaneously with sh-Ctrl or sh-hnRNPA2B1 DLD1 cells. Growth curve (**F**), weight (**G**) and size (**H**) of subcutaneous xenograft tumors were indicated. *n* = 6 biologically independent samples. **I** Representative images of metastatic loci in the lung derived from mouse model that tail vein injected with WT or KO HCT116 cells. Black arrows indicate the tumor nodules on lung surfaces. **J** Representative HE staining of lung metastatic lesion. Scale bar, 500 μm (left×2panel); 20 μm (right×40 panel). **K** Quantification of lung metastatic foci from nude mice. *n* = 5 biologically independent samples. A two-tailed Student’s *t*-test and one-way ANOVA were used for statistical analysis, respectively. ^*^P < 0.05, ^**^P < 0.01, ^***^P < 0.001. Data represent mean ± SD.

To fully understand the clinical significance of hnRNPA2B1 in CRC. The expression level of hnRNPA2B1 in CRC was obtained from the detections of CRC specimens (Fig. S1A, B), cell lines (Fig. S1C, D) and TCGA database (Fig. S1E), which showed that hnRNPA2B1 was significantly upregulated in CRC. Moreover, the patients with deeper tumor invasion depth presented higher hnRNPA2B1 expression (*P* = 0.015) (Table S1). Furthermore, increased expression of hnRNPA2B1 was also detected in CRC TMA by IHC analysis (Fig. S1F, I). According to our TMA data, higher hnRNPA2B1 expression was confirmed to be strongly correlated with more malignant pathological grade (Fig. 1A, *P* = 0.0099), deeper tumor invasion (Fig. S1G, *P* = 0.0440), more severe lymph node metastasis (Fig. S1H, *P* < 0.0001), more advanced TNM stage (Fig. 1B, *P* < 0.0001) (Table 1), and worse survival burden of CRC (Fig. 1C, *P* = 0.0403). Thus, we demonstrated that upregulated hnRNPA2B1 was closely associated with clinicopathological characteristics and survival prognosis in CRC.

**Table 1:** Correlation between hnRNPA2B1 expression and clinicopathological characteristics.

	variables	hnRNPA2/B1 expression		total	*P* value
		low	high		
Age (year)					0.3339
	≤65	19	24	43	
	>65	24	20	44	
Sex					0.9096
	Male	20	21	41	
	Female	23	23	46	
Grade					**0.0099**
	I/II	41	32	73	
	III/IV	2	12	14	
T stage					**0.0440**
	T1-T3	34	26	60	
	T4	9	18	27	
N stage					**<0.0001**
	N0	39	21	60	
	N1/N2	4	23	27	
M stage					0.2414
	M0	43	41	84	
	M1	0	3	3	
TNM stage					**<0.0001**
	I/II	39	19	58	
	III/IV	4	25	29	
Tumor size (cm)					0.2333
	≤5	26	21	47	
	>5	17	23	40	

Bold values indicates statistical significant *P* values (*P* < 0.05).

**Fig. 2:**
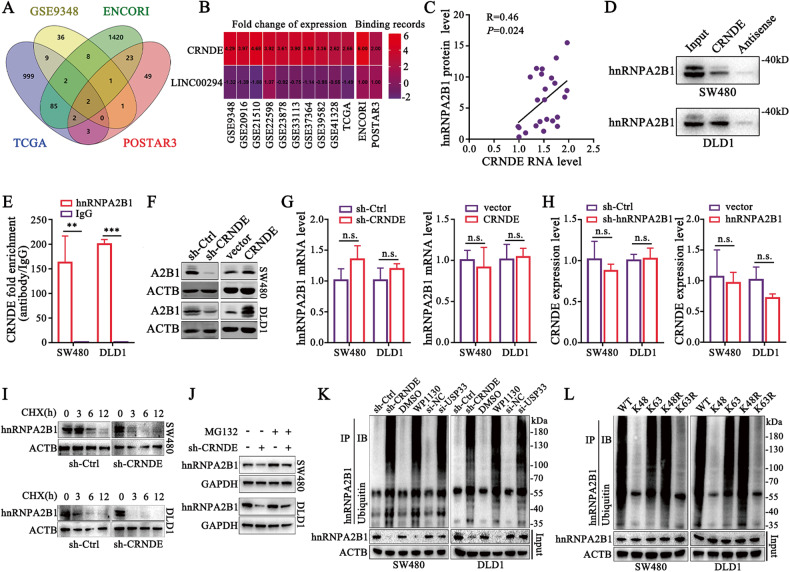
CRNDE binds to hnRNPA2B1 and protects its protein stability in CRC. **A** LncRNAs differentially expressed in CRC were screened in the GSE9348 dataset and TCGA database at *P* < 0.01 and with a fold change>2, which were then intersected with hnRNPA2B1-bound ncRNAs that obtained from the online databases ENCORI and POSTAR3. CRNDE and LINC00294 were enriched. **B** Comparison of the differential expression of CRNDE and LINC00294 in CRC in TCGA and various GEO databases, as well as their binding scores to hnRNPA2B1 in ENCORI and POSTAR3. **C** Correlation analysis between expression levels of hnRNPA2B1 and CRNDE in CRC patients (*R* = 0.46, *P* = 0.024, *n* = 24). **D** RNA pulldown assays were performed using biotin-labeled CRNDE and its antisense, followed by western blot analysis. **E** RIP assays were performed using hnRNPA2B1 specific antibody and IgG, and the co-precipitated RNA was subjected to qRT-PCR to measure the enrichment of CRNDE. *n* = 3 independent biological replicates. **F** Western blot detected the expression changes of hnRNPA2B1 protein in SW480 and DLD1 cells with stable CRNDE knockdown or overexpression. The effects of CRNDE (**G**) and hnRNPA2B1 (**H**) alterations on each other’s RNA levels were examined by qPCR. **I** Western blot detection of hnRNPA2B1 in CRNDE silencing cells treated with CHX (20 μg/mL) for the indicated times. *n* = 3 independent biological replicates. **J** CRNDE knockdown cells that were treated with MG132 (25 μmol/L) for 6 h and then the hnRNPA2B1 levels were tested by western blot. **K** Immunoprecipitated hnRNPA2B1 in extracts of CRC cells with stable CRNDE deletion, DUB inhibitor WP1130 (5 μM for 6 h) treatment, or USP33 knockdown was immunoblotted with an anti-Ubiquitin antibody. WP1130 and si-USP33 were employed as the positive controls. **L** Ubiquitin plasmids with either only K48 or K63 retained, or with only K48 or K63 mutated, along with their wild-type ubiquitin controls, were transfected into CRC cells. Ubiquitin levels in hnRNPA2B1-IP samples were analyzed by immunoblotting. A two-tailed unpaired Student’s *t-*test and Pearson correlation analysis were used for statistical analysis, respectively. ^**^P *<* 0.01, ^***^P < 0.001, n.s. not significantly. Data represent mean ± SD.

To investigate the biological functions of hnRNPA2B1 in CRC, the stably knockdown or overexpressed hnRNPA2B1 cell lines were constructed (Fig. S2A, H). Knockdown of hnRNPA2B1 significantly inhibited CRC cell proliferation, invasion and migration (Fig. S2B–G), while ectopic expression of hnRNPA2B1 dramatically enhanced these malignant behaviors (Fig. S2I–N). Considering that hnRNPA2 and hnRNPB1 differ by 12 amino acids in the N-terminal region, they were suspected of possibly having distinct biological features [26]. We observed that the reintroduction of hnRNPA2 or hnRNPB1 restored the malignant biological behaviors of hnRNPA2B1 knockout (KO) cells, but no statistical difference was found between the two isoforms (Fig. 1D, S3A–C). The similar oncogenic functions of hnRNPA2 and hnRNPB1 in CRC were firstly demonstrated. Consistent with the findings in vitro, we observed that the growth of subcutaneous tumors of nude mice in sh-hnRNPA2B1 group was significantly slower (Fig. 1E, F), lighter (Fig. 2G) and smaller (Fig. 2H) than that in the control group. Meanwhile, the nude mice with KO cells showed a decrease in lung metastasis (Fig. 1I–K). However, no significant differences were captured in the weight of nude mice (Fig. S3D, E). Taken together, these results provided comprehensive evidence that hnRNPA2B1 could significantly contribute to the tumor growth and metastasis of CRC cells in vitro and in vivo, which suggested that hnRNPA2B1 was a clinically crucial oncogene in CRC.

### CRNDE promotes hnRNPA2B1 protein stability

The lncRNA/hnRNP interaction pattern is a key form of regulating various biological programs involved in cancer progression that cannot be ignored. We thus speculated that whether the dysregulation of hnRNPA2B1 in CRC was controlled by lncRNAs. Therefore, the differentially expressed lncRNAs in CRC were analyzed with GEO [27] and TCGA database [28], and the hnRNPA2B1-interacting lncRNAs were identified with ENCORI [29] and POSTAR3 [30]. We turned attention to CRNDE, the only enriched and significantly upregulated lncRNA in several databases (Fig. 2A, B), which has been named as colorectal neoplasia differentially expressed since its discovery and was a well-acknowledged carcinogenic lncRNA in CRC [31]. Moreover, the hnRNPA2B1 protein level was positively correlated with the level of CRNDE (*P* = 0.024) (Fig. 2C). The results of RNA pull-down (Fig. 2D) and the RIP assays (Fig. 2E) unveiled that hnRNPA2B1 indeed bound to CRNDE. Next, based on the predicted secondary structure of CRNDE, a series of CRNDE truncations were constructed (Fig. S4A), and the 401–800nt region of CRNDE was identified as the major interacting domain (Fig. S4B). These results showed that hnRNPA2B1 was a bona fide interacting partner of CRNDE in CRC cells.

Notably, CRNDE was found to positively affect the protein expression of hnRNPA2B1 (Fig. 2F, S4C) rather than mRNA level (Fig. 2G), while hnRNPA2B1 did not cause changes in CRNDE expression (Fig. 2H). We also observed that the protein stability and half-life of hnRNPA2B1 were significantly repressed by CRNDE knockdown (Fig. 2I, S4D). In addition, the decreased hnRNPA2B1 protein was slightly re-upregulated by treatment with the proteasome inhibitor MG132 (Fig. 2J). The ubiquitination of hnRNPA2B1 was significantly enriched after CRNDE knockdown (Fig. 2K). To ascertain that hnRNPA2B1 was modified with polyubiquitination, a set of ubiquitin mutants were used in which either only K63 or K48 Lys residue was retained, or only K63 or K48 Lys residue was mutated, and eventually K63 rather than K48 polyubiquitin was clearly detected (Fig. 2L). Moreover, hnRNPA2B1 was selective polyubiquitinated with K63 but not K48 ubiquitin (Figure S4E). Together, these data indicated that CRNDE protected hnRNPA2B1 from K63 ubiquitin-dependent protein degradation. K63-linked polyubiquitin usually functioned as a regulator of proteins or signaling pathways, instead of mediating proteasome-dependent degradation, but was connected to some self-degradative mechanisms, such as autophagy [32]. In order to explore the specific mechanisms involved, we additionally introduced the specific proteasome inhibitor Bortezomib, as well as the autophagy inhibitors chloroquine and ammonium chloride (NH_4_Cl). Our results revealed that Bortezomib did not affect the K63-linked polyubiquitination of hnRNPA2B1 (Fig. S4F). However, chloroquine and NH_4_Cl obviously blocked the ability of CRNDE knockdown to induce the degradation of hnRNPA2B1, whereas Bortezomib and MG132 were only weakly performed (Fig. S4G). These findings suggested that CRNDE was likely to stabilize hnRNPA2B1 protein level primarily by obstructing the K63 ubiquitin-dependent autophagic degradation pathway.

### CRNDE stabilizes hnRNPA2B1 by preventing TRIM21-mediated ubiquitination

**Fig. 3:**
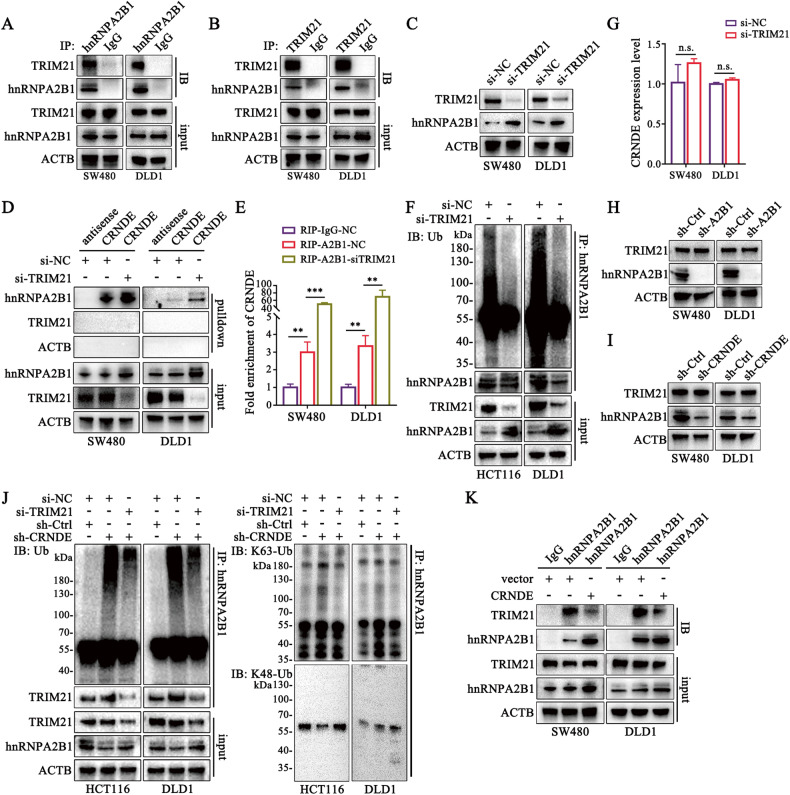
CRNDE stabilizes hnRNPA2B1 by preventing TRIM21-mediated ubiquitination. Co-IP for hnRNPA2B1 (**A**) and TRIM21 (**B**) were performed in indicated CRC cells. IgG was used as negative control. The co-IPs were analyzed by western blot using anti-hnRNPA2B1 or anti-TRIM21 antibodies. **C** The influence of knocking down TRIM21 on the expression levels of hnRNPA2B1 was examined by western blot. The binding of hnRNPA2B1 to CRNDE in protein extracts from indicated cells transiently transfected with si-TRIM21 or si-NC was measured by CRNDE-pulldown (**D**) and hnRNPA2B1-RIP (**E**). *n* = 3 independent biological replicates. **F** Immunoprecipitated endogenous hnRNPA2B1 from cells with TRIM21 depletion was immunoblotted with anti-Ubiquitin antibody. **G** The levels of CRNDE were detected by qPCR after knockdown of TRIM21. *n* = 3 independent biological replicates. Protein expression of TRIM21 detected by western blot after down-regulation of hnRNPA2B1 (**H**) or CRNDE (**I**). **J** TRIM21 was attenuated in CRNDE-silenced cells and IP of hnRNPA2B1 was carried out. Protein changes of total ubiquitin, k63 and K48 ubiquitin, and TRIM21 in immunoprecipitates were identified by western blot, respectively. **K** IP assays were conducted by immobilizing antibodies against hnRNPA2B1 or IgG on Protein A magnetic beads, and the precipitated TRIM21 and hnRNPA2B1 proteins from cell lysates with or without CRNDE overexpression were detected by western blot. A two-tailed unpaired Student’s *t-*test was used for statistical analysis. ^**^P *<* 0.01, ^***^P < 0.001, n.s. not significantly. Data represent mean ± SD.

To further investigate the molecular mechanism of CRNDE stabilized hnRNPA2B1 via K63 polyubiquitination, hnRNPA2B1-immunoprecipitation (IP) and mass spectrometry (MS) were performed (Fig. S5A). The results indicated that TRIM21 was one of the top-ranked E3 ubiquitin ligase (Table S2). We then demonstrated the interactions between hnRNPA2B1 and TRIM21 using endogenous and exogenous co-IP immunoblots (Fig. 3A, B, S5B). We found that TRIM21 knockdown markedly increased the abundance of hnRNPA2B1 (Fig. 3C), and enhanced the interaction of hnRNPA2B1 with CRNDE (Fig. 3D, E), as well as dramatically decreased ubiquitination level of hnRNPA2B1 (Fig. 3F). However, TRIM21 neither bound to CNRDE (Fig. 3D, S5C) nor influence the level of CRNDE (Fig. 3G). Meanwhile, both hnRNPA2B1 and CRNDE did not conversely affect TRIM21 expression either (Fig. 3H, I). Moreover, knockdown of TRIM21 attenuated the elevation of K63 polyubiquitin of hnRNPA2B1 induced by silencing CRNDE (Fig. 3J), and overexpression of TRIM21 remarkably increased K63-linked polyubiquitination of hnRNPA2B1 and decreased the hnRNPA2B1 protein level (Fig. S5D), which indicated that TRIM21 was a key mediator of CRNDE protection of hnRNPA2B1 from K63 ubiquitin-dependent degradation. Additionally, it was worth noting that the enhancement of K63 ubiquitin level of hnRNPA2B1 by TRIM21 was independent of the proteasome system (Fig. S5D). Meanwhile, chloroquine and NH_4_Cl treatments could reverse the effect of TRIM21 on the degradation of hnRNPA2B1 protein, which suggested that the TRIM21-mediated K63 ubiquitin-dependent autophagy pathway might indeed be the primary process regulating hnRNPA2B1 (Fig. S5E). Intriguingly, silencing CRNDE restored the binding between hnRNPA2B1 and TRIM21 (Fig. 3J), while exogenous overexpression of CRNDE attenuated their interactions (Fig. 3K). Therefore, these results strongly demonstrated that CRNDE maintained the protein stability of hnRNPA2B1 by preventing its interaction with TRIM21, and inhibiting TRIM21-mediated K63 polyubiquitination, thereby impeding the process of ubiquitin-dependent protein degradation.

### hnRNPA2B1 is required for CRNDE regulation of CRC cell proliferation and metastasis

**Fig. 4:**
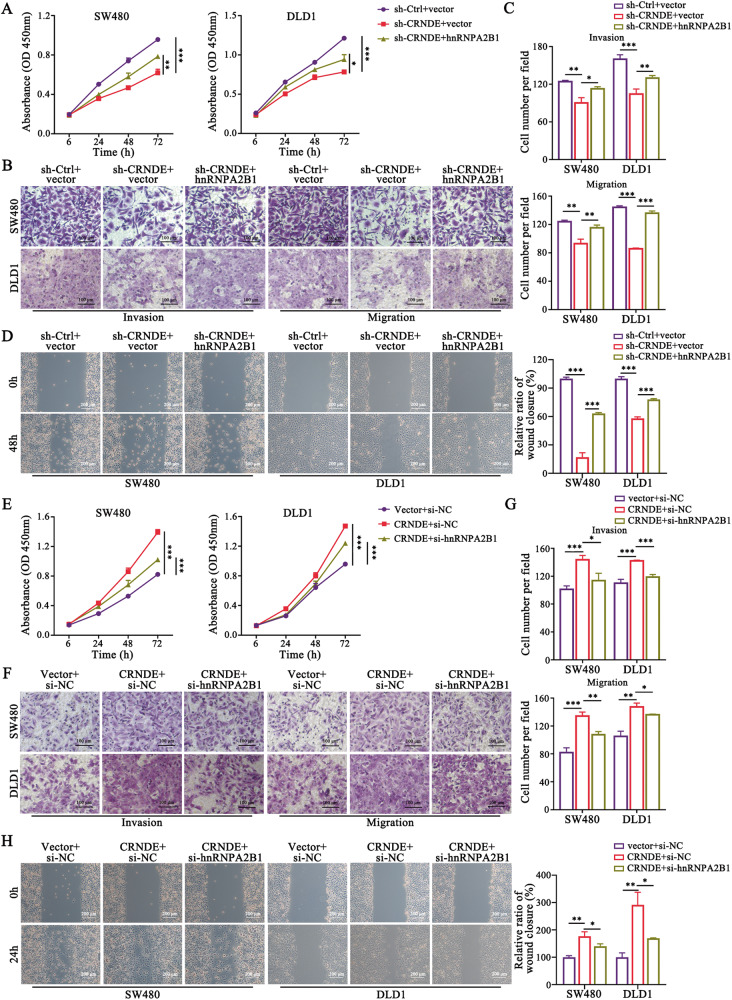
hnRNPA2B1 is required for CRNDE regulation of CRC cell proliferation and metastasis. The hnRNPA2B1 overexpression plasmid or its empty vector was transfected into stable knockdown CRNDE CRC cells SW480 and DLD1 and their proliferative (**A**), invasive and migratory (**B**–**D**) abilities were assessed by functional rescue assays. *n* = 3 independent biological replicates. Scale bar, 100 μm (**B**); 200 μm (**D**). Functional rescue assays were performed in CRNDE overexpression cells with si-hnRNPA2B1 or si-NC transfection to examine the alterations in cell proliferation (**E**) and metastatic (**F**–**H**) capacity. *n* = 3 independent biological replicates. Scale bar, 100 μm (**F**); 200 μm (**H**). A two-tailed unpaired Student’s *t-*test and one-way ANOVA were used for statistical analysis, respectively. ^*^P *<* 0.05, ^**^P *<* 0.01, ^***^P < 0.001. Data represent mean ± SD.

To further elucidate the effect of CRNDE/hnRNPA2B1 in CRC progression, we firstly confirmed the specific upregulation of CRNDE in CRC (Fig. S5F) and its negative correlation with favorable prognosis (Fig. S5G). Subsequently, we found that the inhibitory effect of CRC cell proliferation and metastasis resulting from CRNDE knockdown was reversed by hnRNPA2B1 upregulation (Fig. 4A–D), and the depletion of hnRNPA2B1 weakened the promotion of CRC malignant biological properties caused by increased CRNDE (Fig. 4E–H). These data suggested that hnRNPA2B1 was a critical link in the function of CRNDE and the CRNDE/hnRNPA2B1 axis was an important driver of accelerating CRC progression.

### CRNDE/hnRNPA2B1 axis regulates MAPK pathway by targeting KRAS

**Fig. 5:**
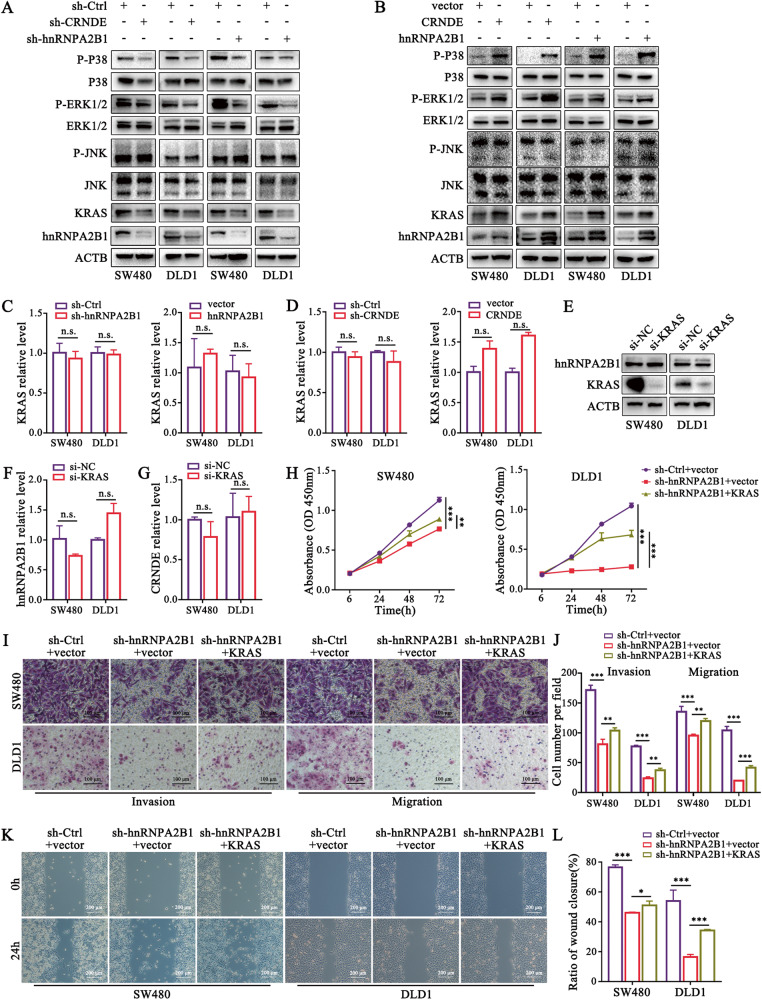
CRNDE/hnRNPA2B1 axis targets KRAS and activates MAPK pathway in CRC. Western blot analysis for the expression changes of MAPK signaling pathway-related marker P38, ERK1/2, JNK and their corresponding phosphorylated proteins in SW480 and DLD1 cells with stably silencing or overexpressing hnRNPA2B1 (**A**) or CRNDE (**B**). **C**, **D** qPCR measure of changes in KRAS mRNA levels following alterations in hnRNPA2B1 or CRNDE. *n* = 3 independent biological replicates. **E** Detection of hnRNPA2B1 protein expression by western blot in CRC cells after knockdown of KRAS. **F**-**G** Quantification of hnRNPA2B1 (**F**) and CRNDE (**G**) RNA levels in KRAS-silenced CRC cells by qPCR, respectively. *n* = 3 independent biological replicates. **H**–**L** Transfection of KRAS overexpression plasmids and their controls into stably silenced hnRNPA2B1 cells for functional rescue experiments. *n* = 3 independent biological replicates. Scale bar, 100 μm (**I**); 200μm (**K**). A two-tailed unpaired Student’s *t-*test and one-way ANOVA were used for statistical analysis, respectively. ^*^P *<* 0.05, ^**^P *<* 0.01, ^***^P < 0.001. n.s. not significantly. Data represent mean ± SD.

Previous studies have reported that hnRNPA2B1 was inextricably associated with the MAPK signaling pathway in cancer development [15, 22]. Moreover, KRAS, the core upstream regulator of the MAPK pathway [33], has recently been found to be interacted with hnRNPA2B1 in PDAC cells, but the exact mechanisms underlying hnRNPA2B1-mediated KRAS-MAPK remain to be elucidated. We found that the protein level of KRAS, p-ERK1/2 and p-P38 was decreased after inhibition of CRNDE and hnRNPA2B1 (Fig. 5A), and conversely increased with their elevation (Fig. 5B), which indicated that the KRAS/MAPK pathway was the downstream signaling cascade of CRNDE/hnRNPA2B1 axis. However, it is noteworthy that neither hnRNPA2B1 nor CRNDE altered KRAS mRNA levels (Fig. 5C, D), and KRAS knockdown did not affect the expression of hnRNPA2B1 and CRNDE in turn (Fig. 5E–G). These results demonstrated that hnRNPA2B1 regulated KRAS at the post-transcriptional level.

Furthermore, the ectopic expression of KRAS impaired the inhibitory effect of hnRNPA2B1 depletion on proliferation, invasion and migration of CRC cells (Fig. 5H–L). To clarify whether the role of CRNDE/hnRNPA2B1/KRAS axis in CRC is KRAS mutation-dependent, we introduced the KRAS wild-type CRC cell line RKO and HT29. The results showed that silencing hnRNPA2B1 and CRNDE similarly downregulated KRAS protein levels in RKO and HT29 (Fig. S6A, D), and inhibited cell proliferation and metastasis (Fig. S6B, C, S6E, F). These results suggested that the function of CRNDE/hnRNPA2B1 axis controlled MAPK pathway by regulating KRAS protein expression, which was not dependent on KRAS mutations.

### CRNDE cooperates with hnRNPA2B1 to facilitate KRAS mRNA nuclear export and translation

**Fig. 6:**
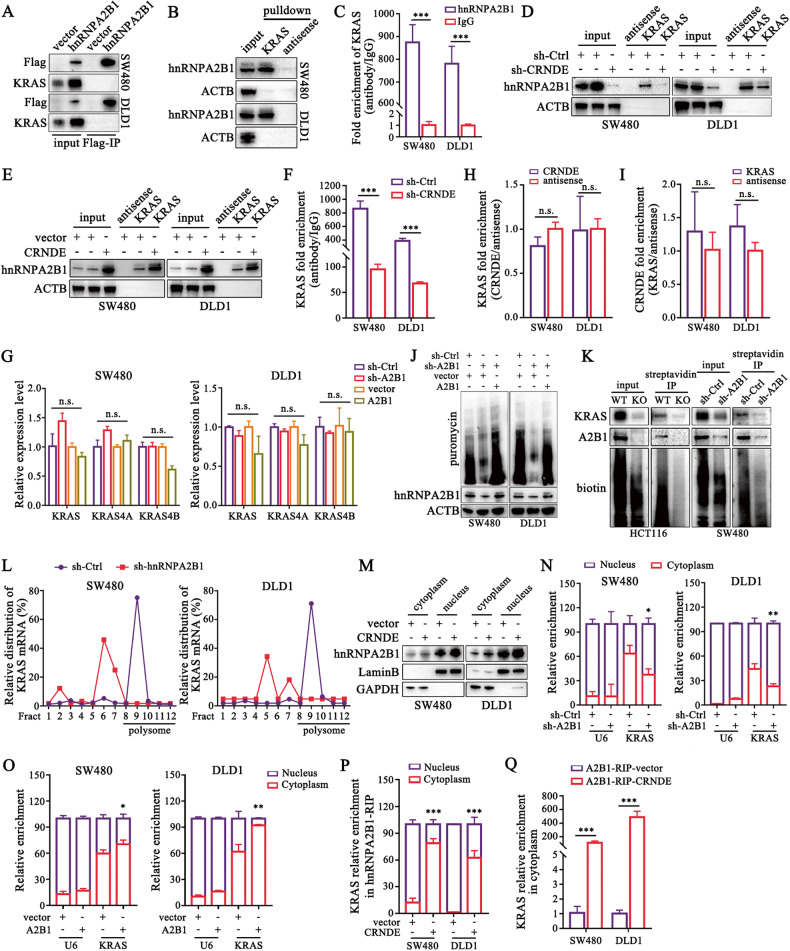
CRNDE cooperates with hnRNPA2B1 to promote KRAS mRNA nuclear export and translation. **A** Flag-IP assays were performed in CRC cells with Flag-tagged hnRNPA2B1 overexpression plasmids transfection. **B** KRAS-pulldown assays were conducted and then were analyzed by western blot. **C** hnRNPA2B1-RIP-qPCR assays were undertaken to examine the enrichment of KRAS mRNA. KRAS-pulldown assays were performed in cells stably silenced (**D**) or overexpressing (**E**) CRNDE to evaluate the altered binding efficiency of hnRNPA2B1. **F** The hnRNPA2B1-RIP assays were executed after knockdown of CRNDE. **G** qPCR detection of KRAS variant KRAS4A and KRAS4B. **H**, **I** The pulldown products of CRNDE and KRAS were identified by qPCR. **J** SUnSET assays were employed to detect the puromycin-labeled proteins in CRC cells with decreasing or increasing of hnRNPA2B1 followed by treatment with 10 μg/mL puromycin for 15 min. **K** hnRNPA2B1-deficient cells with Biotin-dC-puromycin treatment for 24 h were collected for precipitation with streptavidin beads, and the products were measured by western blot. **L** The ribosomal fractions were separated on a sucrose gradient and their RNA was extracted. Amount of KRAS mRNA was analyzed by qRT-PCR. **M** Western blot detection of hnRNPA2B1 levels in the nuclear and cytoplasmic fractions of CRC cells stably overexpressing CRNDE. **N**–**O** Cytoplasm and nucleus of CRC cells with stable hnRNPA2B1 knockdown and overexpression were isolated, of which KRAS mRNA levels were subsequently detected via qPCR. **P**, **Q** Cytoplasm and nuclei of CRC cells with CRNDE upregulation were isolated and extracted, and then hnRNPA2B1-RIP-qPCR analysis for KRAS enrichment were performed to determine the effect of CRNDE on the distribution and efficiency of hnRNPA2B1-KRAS mRNA interaction. *n* = 3 independent biological replicates. A two-tailed unpaired Student’s *t-*test and one-way ANOVA were used for statistical analysis, respectively. ^*^P *<* 0.05, ^**^P *<* 0.01, ^***^P < 0.001. n.s. not significantly. Data represent mean ± SD.

We next aimed to clarify the post-transcriptional mechanisms by which hnRNPA2B1 affected KRAS. Previously, hnRNPA2B1 was reported to interact directly with KRAS proteins as a partner without regulating KRAS expression in pancreatic ductal adenocarcinoma (PDAC) [33]. However, we did not detect any KRAS protein signal in the immunoprecipitated samples of Flag-hnRNPA2B1 (Fig. 6A), implying that hnRNPA2B1 might carry distinct functional missions in different cancer species. Since hnRNPA2B1 is a well-recognized classical RBP that is a key driver switch for target molecule splicing, translocation and translation, we sought to investigate whether hnRNPA2B1 regulated the translation of bound KRAS mRNA.

Firstly, we confirmed the protein-RNA binding relationship between hnRNPA2B1 and KRAS (Fig. 6B, C). Subsequently, knockdown or overexpression of CRNDE, respectively, revealed that the binding between hnRNPA2B1 and KRAS was weakened (Fig. 6D, F) or strengthened (Fig. 6E). Moreover, hnRNPA2B1 or CRNDE did not regulate KRAS splicing (Fig. 6G, S7A). Meanwhile, we also found that there was no binding between CRNDE and KRAS RNA (Fig. 6H, I). Based on these results we hypothesized that CRNDE/hnRNPA2B1 axis promoted KRAS protein expression by translational control of KRAS mRNA.

To gain further insight into its mechanism, the SUnSET assays were employed, and the results unveiled that hnRNPA2B1 strongly influenced global protein synthesis (Fig. 6J, S7B). Next, puromycin-labeling assays were utilized to monitor the synthesis of nascent KRAS protein and, as expected, a significant reduction in the puromycin labeling of KRAS was shown following hnRNPA2B1 deletion (Fig. 6K). Furthermore, we used polysome profiling and qRT-PCR to examine the distribution of endogenous hnRNPA2B1-targeted KRAS mRNA in the ribosome fractions to quantify the proportion that was translated. We found that the relative distribution of KRAS mRNA shifted from the polysome to the subpolysome fraction in hnRNPA2B1 silencing cells (Fig. 6L). These results demonstrated that hnRNPA2B1 facilitated the translation of KRAS mRNA.

Intriguingly, we unexpectedly detected that CRNDE promoted hnRNPA2B1 nucleocytoplasmic translocation (Fig. 6M). We thus speculated that hnRNPA2B1 regulated nuclear transport of KRAS mRNA and subsequently provided translational control of KRAS. The mRNA distribution of KRAS following hnRNPA2B1 depletion or elevation was then examined, and the results showed that knockdown of hnRNPA2B1 reduced cytoplasmic aggregation of KRAS mRNA (Fig. 6N), while conversely overexpression of hnRNPA2B1 boosted KRAS mRNA nuclear export (Fig. 6O). Concurrently, we observed that overexpression of CRNDE expedited hnRNPA2B1-KRAS complex translocation to cytoplasm (Fig. 6P–Q). Collectively, our data indicated that CRNDE/hnRNPA2B1 axis enhanced nuclear export and its subsequent translational control of KRAS mRNA in CRC cells.

## Discussion

CRC involves aberrant expression of numerous gene regulatory networks, making it a struggle to understand and attack this most common and deadly of human malignancies at the forefront [34, 35]. However, the critical molecules modulating CRC progression are still largely unknown. Here, we revealed a model for nuclear export and translational control of CRDNE/hnRNPA2B1 oncogenic axis-mediated KRAS expression in CRC cells and identified a novel E3 ligase TRIM21 in regulating K63 ubiquitin-dependent degradation of hnRNPA2B1.

hnRNPA2B1 plays a vital role in a variety of diseases including amyotrophic lateral sclerosis [36, 37], pulmonary arterial hypertension [38], obesity [39] and innate immune response [40], particularly in cancer, where hnRNPA2B1 has been recommended as a candidate marker for cancer screening [7]. hnRNPA2B1 was identified to distinguish early-stage lung cancer with sensitivity of 84.8% in brushing, 80.8% in biopsies and 72.2% in serum [41, 42]. Interestingly, the intracellular localization of hnRNPA2B1 was found to alter during the transition from hepatitis virus infection to poorly differentiated hepatocellular carcinoma [43, 44], and its nucleoplasmic shift might be related to cell differentiation [45]. In addition, upregulated hnRNPA2B1 is also associated with the development of glioblastoma [46], pancreatic cancer [11], multiple myeloma [47], prostate cancer [48], ovarian cancer [49] and CRC [14]. However, the mechanism of upregulating hnRNPA2B1 expression has not yet been fully elucidated.

The lncRNA/hnRNPA2B1 interaction network has been proposed to play a non-negligible role in CRC, but none of these studies illuminated the upregulation of hnRNPA2B1. Some studies have reported the transcriptional and post-transcriptional regulation of hnRNPA2B1 in cancer, that c-Myc [50] and BRCA1 [51] promoted hnRNPA2B1 transcription, while activation of Fyn [52] and Nm23-H1 [53] stabilized hnRNPA2B1 protein expression. Several lncRNAs were also identified to modulate the ubiquitin-dependent degradation of hnRNPA2B1, such as uc002mbe.2 [54] and miR503HG [12] to accelerate its degradation, and conversely Linc01232 [11] protected hnRNPA2B1 from ubiquitin-mediated proteasomal degradation. VHL is currently the only known E3 ligase to mediate ubiquitination of hnRNPA2B1 in renal cancer cells [55, 56], but we did not find interactions of them in CRC cells (data not shown). Therefore, there are no convincible answers to explain the mechanism of hnRNPA2B1 protein upregulation. In this study, we recognized that CRNDE was the only candidate lncRNA, which stabilized hnRNPA2B1 protein. CRNDE, an oncogenic lncRNA originally identified for the first time in CRC, possesses a pivotal role in the biological process of CRC [57]. Moreover, we also identified a new E3 ubiquitin ligase TRIM21 of hnRNPA2B1 by co-IP-MS, which promoted K63-linked ubiquitin-dependent degradation of hnRNPA2B1. K63-linked ubiquitination was also functioned in autophagic degradation [32]. Additionally, TRIM21 was discovered to interact with autophagy regulators and effectors, which then promoted autophagic degradation of targeted proteins [58]. Moreover, TRIM21 was decreased in colitis-associated cancer and inhibited intestinal epithelial carcinogenesis [59]. Meanwhile, hnRNPA2B1 was detected as co-localized with selective autophagy receptor SQSTM1 [59, 60]. Therefore, based on our experimental findings, we hypothesized that CRNDE might have a partial impact on the ubiquitin-proteasome system of hnRNPA2B1, but the hnRNPA2B1 expression was primarily regulated by TRIM21-mediated K63 ubiquitin-dependent autophagic degradation pathway. Nevertheless, further investigation is required to fully elucidate this mechanism.

KRAS/MAPK oncogenic signaling plays a central role in CRC progression. A protein interaction between hnRNPA2B1 and KRAS has been reported in PDAC cells [33], but we did not recapitulate this pattern in CRC cells. Furthermore, both CRNDE and hnRNPA2B1 have been suggested to be activators of the MAPK signaling pathway. However, the mechanism by which the MAPK signaling pathway is regulated by the CRNDE/hnRNPA2B1 axis remains inconclusive. Here, we demonstrated that CRNDE/hnRNPA2B1 axis drove CRC development through activation of KRAS/MAPK signals. Intriguingly, we found that the CRNDE/hnRNPA2B1 axis controlled KRAS protein, but did not affect KRAS mRNA expression or splicing, suggesting that hnRNPA2B1 regulated KRAS protein expression at the post-transcriptional level.

hnRNPA2B1 could promote cap-dependent translation of RNA trafficking signal sequence from myelin basic protein mRNA [61], and also could bind Sp1 [53], ABCC2 [62], nmMYLK [63], HIF-1α [64] and VHLα [56] mRNA to enhance their translation, respectively. The translation of KRAS mRNA was also influenced by eIF4A [65], RNA G-quadruplexes [66] or eIF5A-PEAK1 loop [67]. We then sought to investigate whether hnRNPA2B1 governed the translation of bound KRAS mRNA. In consistence with our hypothesis, our results confirmed that hnRNPA2B1 protein was directly bound to and contributed to the translation of KRAS mRNA. In addition, upregulation of CRNDE enhanced the binding capacity between them, but there was no physical interaction of CRNDE with KRAS RNA. Unexpectedly, we also found that overexpression of CRNDE facilitated nucleocytoplasmic transport of hnRNPA2B1. Given the importance of aberrant expression and localization of hnRNPA2B1 in digestive cancer [43, 45], and the powerful ability of hnRNPA2B1 to regulate RNA trafficking [68, 69], we subsequently demonstrated that hnRNPA2B1 enhanced KRAS nuclear export and that upregulated CRNDE potently encouraged the cytoplasmic accumulation of the hnRNPA2B1-KRAS complex. Collectively, our findings unveiled that the CRNDE/hnRNPA2B1 axis drove the coupled mRNA transport-translation processes in CRC cells. However, the partners of hnRNPA2B1 in regulating mRNA nucleocytoplasmic transport and translation need further investigation.

In summary, our paper elucidated that upregulation of CRNDE enhanced hnRNPA2B1 protein stability via inhibiting TRIM21-mediated K63 ubiquitin-dependent degradation, and then promoted nuclear export and translation of KRAS mRNA, which subsequently activated MAPK oncogenic signaling in CRC cells. Thus, our study revealed the CRNDE/hnRNPA2B1/KARS axis played a critical driving role in CRC malignant progression, reserving a promising prognostic biomarker and potential therapeutic target for CRC.

## Supplementary information

Supplementary Information

Original western blots

## Data Availability

All data used in this work can be acquired from the TCGA database (http://gepia.cancer-pku.cn), ENCORI (https://starbase.sysu.edu.cn), POSTAR3 (http://postar.ncrnalab.org), and Gene Expression Omnibus (GEO) datasets. The processed data are available from the corresponding author on reasonable request.
