# Supplementary material for: CRNDE mediated hnRNPA2B1 stability facilitates nuclear export and translation of KRAS in colorectal cancer
Source: Cell Death Dis. 2023 Sep 16;14(9):611. doi: 10.1038/s41419-023-06137-9 (PMC10505224; doi:10.1038/s41419-023-06137-9)
Supplement: Supplementary file 1 — Supplementary Information [file 41419_2023_6137_MOESM1_ESM.doc]

**Supplementary Materials and Methods**

**Cell culture**

The human CRC cell lines DLD1, SW480, SW620, HCT116, HT29 and RKO as well as the human colonic epithelial cell line HCoEpiC were purchased from the American Type Culture Collection and cultured in Dulbecco's Modified Eagle Medium (DMEM, KeyGEN BioTECH, Jiangsu, China) containing 100 U/ml penicillin and 100 μg/ml streptomycin and supplemented with 10% fetal bovine serum (FBS, Gibco, CA, USA). All cell lines were maintained at 37°C in a humidified incubator with 5% CO_2_. All these cells were periodically tested for mycoplasma contamination.

**RNA isolation and qRT-PCR analysis**

Total RNA in cell lines was isolated by using EZ-10 DNAaway RNA Mini-Preps Kit (Sangon Biotech, Shanghai, China) and reverse transcribed into cDNA with PrimeScript RT Master Mix (TaKaRa, Dalian, China) following the instruction manual. qPCR analysis was performed using PowerUp™ SYBR Green Mix (ThermoFisher, CA, USA) and ABI Prism 7500 Real-Time PCR System (Applied Biosystems, CA,USA). The relative level of mRNA in cell lines was normalized to that of GAPDH and calculated with the 2^−ΔΔ^**^Ct^** method, while mRNA relative level in tissues was normalized to 18S rRNA and assessed with the 2^−ΔCt^ method. No-RT control was set up in each set of experiment forcontaminant DNA. Sequences of the primers used for PCR analysis were listed in Table S3.

**Western blot and antibodies**

Protein lysates from cells or tissues were extracted with RIPA buffer (ThermoFisher, CA, USA) supplemented with protease and phosphatase inhibitors (NCM Biotech, Suzhou, China). Nucleus and cytoplasm proteins were isolated by the Nuclear and Cytoplasmic Protein Extraction Kit (KeyGEN BioTECH, Nanjing, China) according to the manufacturer’s instruction. The protein concentration was quantified by BCA Protein Assay Kit (ThermoFisher, CA, USA). Equal protein lysates were separated by pre-made 4-12% gradient SDS-PAGE (GenScript ProBio, Nanjing, China), transferred onto 0.45 μm PVDF membranes (Milipore, MA, USA), and then blocked with 5% skim milk for 1 h at room temperature. The membranes were then incubated with specific primary antibodies overnight at 4℃. The following primary antibodies hnRNPA2B1 (sc-374053; Santa Cruz, CA, USA), ACTB (AC006; ABclonal, Wuhan, China), FLAG (#D6W5B; Cell Signaling Technology (CST), MA, USA), Ubiquitin (sc-8017; Santa Cruz), K63-linkage Polyubiquitin (A18164; ABclonal), K48-linkage Polyubiquitin (A18163; ABclonal), TRIM21 (12108-1-AP; Proteintech, Wuhan, China), KRAS (12063-1-AP; Proteintech), ERK1/2 (#9194; CST), p-ERK1/2 (#9101; CST), p38 (#8690; CST), p-p38 (#4511; CST), GAPDH (AC002; ABclonal) and LaminB1 (12987-1-AP; Proteintech) were used in this study. After being washed for three times, the membranes were then incubated with the corresponding secondary antibodies (1:5000; Santa Cruz). Protein bands were visualized by ECL chemiluminescence reagent (NCM Biotech, Suzhou, China) and Odyssey imaging system (LI-COR Biosciences).

**Transient transfection**

The small interference RNAs (siRNAs) of hnRNPA2B1, CRNDE, TRIM21, KRAS, USP33 and negative control (NC) were obtained from RiboBio (Guangzhou, China). The Flag-tagged overexpression plasmid of hnRNPA2, hnRNPB1 and KRAS, and corresponding empty vector pLenti-CMV-GFP-Puro were obtained from Public Protein/Plasmid Library (PPL, Jiangsu, China). HA-tagged ubiquitin plasmids (pCMV-HA-UB-K63, pCMV-HA-UB-K48, pCMV-HA-Ub-K63R, pCMV-HA-Ub-K48R, and pCMV-HA-Ub) were obtained from MiaoLing Plasmid Platform (Wuhan, China). Flag-tagged hnRNPA2B1 and HA-tagged TRIM21 were obtained from SinoBiological (Beijing, China). The plasmid DNA or siRNAs were transiently transfected into cells with Lipofectamine 3000 (Invitrogen, CA, USA) according to the manufacturer's recommendations. The sequences of siRNAs were provided in Table S4.

**Generation of stable CRC cell lines**

CRC SW480 and DLD1 cells were infected with puromycin-resistant lentiviruses that knockdown or overexpress hnRNPA2B1 or CRNDE. These specific lentiviruses and their corresponding controls were obtained from Corues Biotechnology (Jiangsu, China). The stable CRC cell lines were established by puromycin screening. The hnRNPA2B1 knockout cell line (KO) used in this study was generated by Corues Biotechnology (Jiangsu, China) in HCT116 using CRISPR-case9 genome engineering technology and identified by sequencing. Western blot and qRT-PCR analysis were applied to identify the efficiency of the stable CRC cell lines construction.

**Cell counting Kit-8 (CCK-8) assay and EdU analysis**

Cell proliferation was assessed using CCK-8 reagent (Dojindo, Japan) and the 5-ethynyl-2’-deoxyuridine (EdU) kit (RIBOBIO, Guangzhou, China) according to the manufacturer’s instructions. Briefly, for CCK-8 assays, 4×10^3^ cells were seeded into 96-well plates, and cell viability was then assessed by OD values at 450 nm when cells adhered and every 24 h thereafter. As for EdU analysis, images were captured and the fraction of EdU-positive cells was calculated with a fluorescence microscope (Olympus Corporation, Tokyo, Japan).

**Wound healing assay**

The cells were cultured in 6-well plates, and scratched wounds were made using 10-µL sterile tips when the cells exceeded 90% density. The extent of wound closure was observed and quantified by taking images under the optical microscope after 0 and 24/48 h.

**Migration and invasion transwell assay**

Cell migration and invasion transwell assays were performed using 8-mm pores transwell chambers (Corning, NY, USA) precoated without (migration assay) or with (invasion assay) Matrigel (Corning, NY, USA). Approximately 4×10^4^ cells in 200 µL serum-free medium were plated into the each upper chamber of each insert, and the lower chambers were filled with 10% FBS containing medium. After 48 hours of incubation, the cells on the filter surface were photographed and counted after being fixed with 4% paraformaldehyde and stained with Crystal Violet Solution (Beyotime Biotechnology, Shanghai, China).

**Animal experiment**

BALB/c nude mice (4-6 weeks of age) were obtained from Charles River Laboratory (Beijing, China) and handled in accordance with the instructions approved by the Committee on the Ethics of Animal Experiments of Nanjing Medical University (No. IACUC-2006034). Taking into account the effect size and standard deviation, the sample size for the animal study was determined following the recommendations of the Animal Ethics Committee in order to ensure the establishment of well-behaved animal models would possess adequate statistical power. For the xenograft tumor models, mice were randomly divided into two groups (sh-ctrl/sh-hnRNPA2B1, n=6), approximately 6×10^6^ cells in each group were subcutaneously injected into the nude mice respectively. The tumor growth was monitored every 4 days and tumor volumes were calculated according to the equation 0.5×length×wildth^2^. The mice were sacrificed at 5-6 weeks after injection and the subcutaneous tumors were measured and used for further analysis. For tail vein metastasis models, a total of 10 nude mice were randomized into two groups (WT or KO), and then injected via the tail vein with 5×10^5^ corresponding cells (100 μL). When the mice reached plateau weight, they were euthanized, and the lung metastases were quantified and analyzed. The investigator was not blinded in animal study.

**RNA pull-down assay**

RNA pull-down assays were performed using Pierce Magnetic RNA-Protein Pull-down Kit (ThermoFisher, CA, USA) according to the manufacturer's instructions. Briefly, the full-length or truncated fragments of KRAS or CRNDE sequence was biotin-labeled and transcribed in vitro using Biotin RNA Labeling Mix and T7 RNA Polymerase Kit (Ribobio, Guangzhou, China) following the manufacturer's protocols. The retrieved proteins that eluted from RNA-interacting complex were identified by western blot.

**Immunoprecipitation (IP) assay** **and mass spectrometry analysis**

IP assays were performed using Dynabead Protein G IP Kit (Thermo Fisher Scientific Inc., NY, USA), Pierce Anti-DYKDDDDK Magnetic Agarose (Thermo Fisher Scientific Inc., NY, USA) or Pierce Anti-HA Magnetic Beads (Thermo Fisher Scientific Inc., NY, USA) as described by the manufacturer. When IP assays related to ubiquitination were conducted, cells were pre-treated with 25 μM proteasome inhibitor MG132 for 6 h. The immunocomplexes were subjected to mass spectrometry (MS) or western blot.

**Protein half-life assay**

The stable cells with CRNDE knockdown were treated with 20 μg/mL protein synthesis inhibitor cycloheximide (CHX) for the indicated times, and then the protein lysates were isolated to detected the level of hnRNPA2B1 by western blot.

**Isolation of RNA from nuclear and cytoplasmic fractions**

Nuclear and cytoplasmic RNA was isolated with the PARIS Kit (Ambion, Life Technologies, Carlsbad, CA, USA) according to the manufacture’s protocol. Cells were lysed in ice-cold cell fraction buffer for 10 min and then placed in centrifugation at 500 g for 5 min at 4°C to separate the cytoplasmic supernatant and nuclear precipitation. RNA from the corresponding fraction was extracted by TRIzol reagent (Invitrogen, CA, USA) and subsequently analyzed by qRT-PCR.

**Puromycin labeling assay**

To detect the change of nascent KRAS synthesis, puromycin labeling assays were performed according to previous procedures. Cells in logarithmic growth phase were incubated with Biotin-dC-puromycin (Jena Bioscience, Heidelberg, Germany) at 1:1000 for 24 h. Cells were then lysed with 1% NP-40 buffer containing proteasome inhibitor cocktail. Afterwards, the supernatant was incubated with streptavidin sepharose beads (Thermo Fisher Scientific Inc., NY, USA) by rotating at 4°C overnight. After completion of incubation the mixture was assayed using western blot.

**Polysome** **fractionation**

Polysome fractionation separated translated mRNAs on a sucrose gradient according to the number of bound ribosomes as previously described. Briefly, cells were treated with 100 μg/mL CHX for 15 min, followed by lysis on ice with polysome buffer for 15 min. The lysates were collected by centrifugation at 12,000g for 15 min at 4°C and then loaded on top of a 10-50% sucrose gradient with ultracentrifugation at 39,000 rpm for 2.5 h at 4°C. The RNA in each fraction was extracted using TRIzol reagent (Invitrogen, CA, USA) and the translational status of a given mRNA species was analyzed by qRT-PCR amplification.

**References**

1. Schneider-Poetsch T, Ju J, Eyler DE, Dang Y, Bhat S, Merrick WC, et al. Inhibition of eukaryotic translation elongation by cycloheximide and lactimidomycin. *Nat Chem Biol* **6**, 209-217 (2010).

2. Aviner R, Geiger T & Elroy-Stein O. Genome-wide identification and quantification of protein synthesis in cultured cells and whole tissues by puromycin-associated nascent chain proteomics (PUNCH-P). *Nat Protoc* **9**, 751-760 (2014).

3. Zhang Y, Wang X, Zhang X, Wang J, Ma Y, Zhang L, et al. RNA-binding protein YTHDF3 suppresses interferon-dependent antiviral responses by promoting FOXO3 translation. *Proc Natl Acad Sci U S A* **116**, 976-981 (2019).

**Supplementary figures**

**
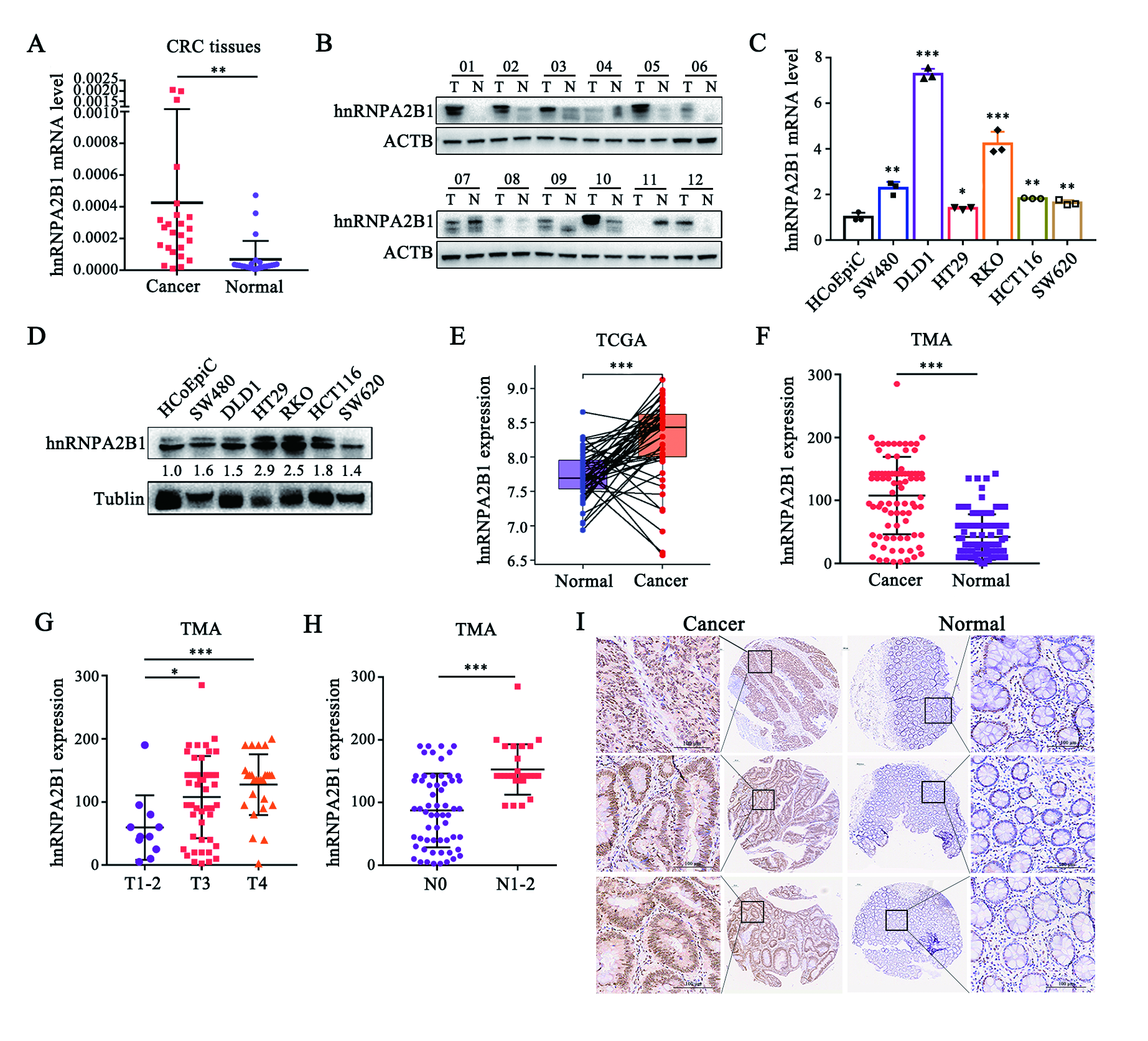
**

**Figure S1** High expression of hnRNPA2B1 in CRC is associated with poor prognosis. **A** qPCR detection of hnRNPA2B1 expression in CRC cancer-normal tissues (n=24). **B** Western blot analysis to detect hnRNPA2B1 expression in CRC tissues and their paracancerous tissues. **C-D** The expression of hnRNPA2B1 in CRC cell lines was measured by qPCR (C) and western blot (D), respectively. Normal intestinal epithelial cells HCoEpiC were used as control. **E** Expression of hnRNPA2B1 in CRC according to TCGA database. **F** Comparison of hnRNPA2B1 expression in CRC and normal colorectal tissues via TMA (n=168). **G-H** Correlation analysis between hnRNPA2B1 and T stage (G) and N stage (H) based on TMA data. **I** Representative IHC staining of hnRNPA2B1 in CRC TMA. Scale bar, 100μm. A two-tailed Student’s t-test and one-way ANOVA were used for statistical analysis, respectively. **P*＜0.05, ***P*＜0.01, ****P*<0.001. Data represent mean±SD.


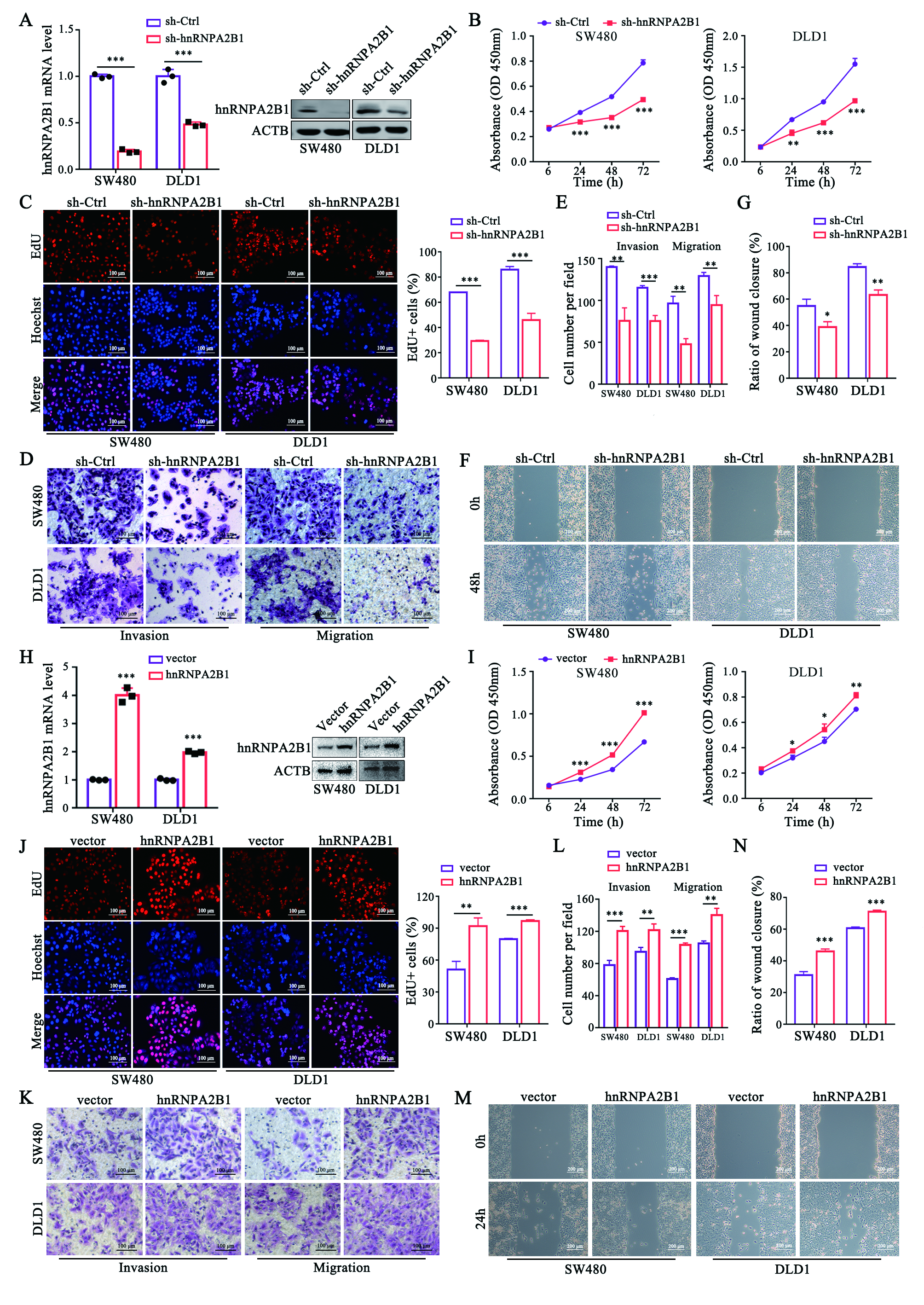


**Figure S2** hnRNPA2B1 promotes CRC cell proliferation, invasion and migration. **A** qPCR (left panel) and western blot (right panel) to validate the efficiency of stable knockdown of hnRNPA2B1 constructed in SW480 and DLD1. **B-C** Knockdown of hnRNPA2B1 suppressed the proliferation capacity of CRC cells, as measured by CCK8 (B) and EdU (C) assays. **D-G** The invasion and migration of hnRNPA2B1-silenced CRC cells was assessed and quantified by Transwell (D, E) and wound healing assays (F, G). **H** The efficiency of stable overexpression of hnRNPA2B1 was examined by qPCR (left panel) and western blot (right panel). **I-J** CCK8 (I) and EdU (J) assays showed that hnRNPA2B1 overexpression increased the ability of CRC cell proliferation. **K-N** Transwell (K, L) and wound healing assays (M, N) exhibited that elevated hnRNPA2B1 enhanced the invasive and migratory capacity of CRC cells. n=3 independent biological replicates. A two-tailed unpaired Student’s *t-*test and one-way ANOVA were used for statistical analysis, respectively. **P＜*0.05, ***P＜*0.01, ****P*<0.001. Data represent mean±SD.

**
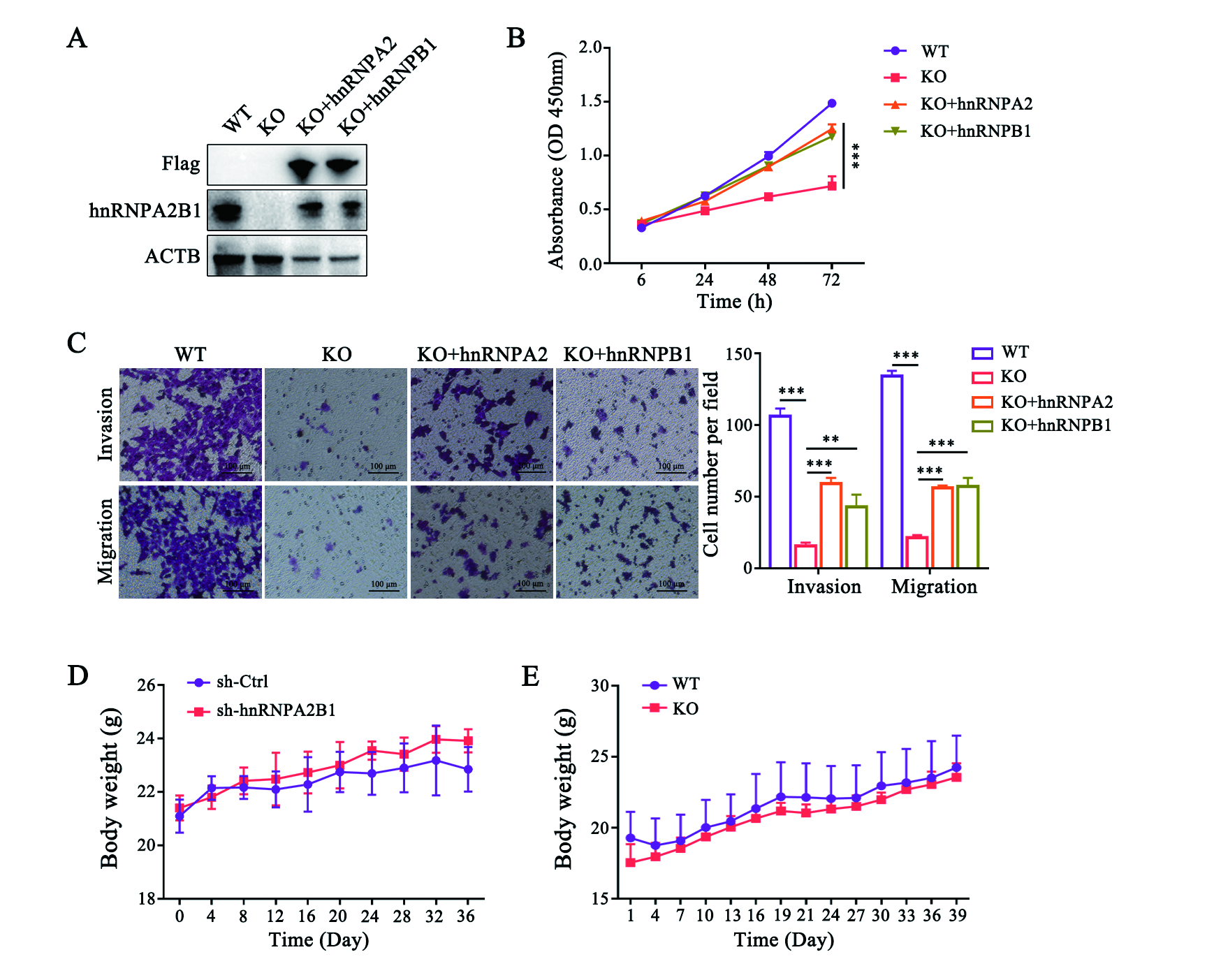
**

**Figure S3** hnRNPA2B1 promotes CRC progression without differences in transcripts. **A** Western blot analysis of hnRNPA2B1 protein expression in hnRNPA2B1-knock out cells transfected with hnRNPA2 and hnRNPB1 overexpression plasmids containing the flag tag. **B-C** The roles of hnRNPA2 and hnRNPB1 variants on the proliferative capacity of CRC cells were measured and quantified by CCK8 assays (B), and the changes in cell invasion and migration ability were detected by Transwell assays (C). Scale bar, 100 μm. n=3 independent biological replicates. **D-E** Body weight change curves of mice from the subcutaneous xenograft tumour models (D) (n=6 biologically independent samples) and the tail vein metastasis tumor models (E) (n=5 biologically independent samples). A two-tailed unpaired Student’s *t-*test and one-way ANOVA were used for statistical analysis, respectively. ***P＜*0.01, ****P*<0.001. n.s., not significantly. Data represent mean±SD.


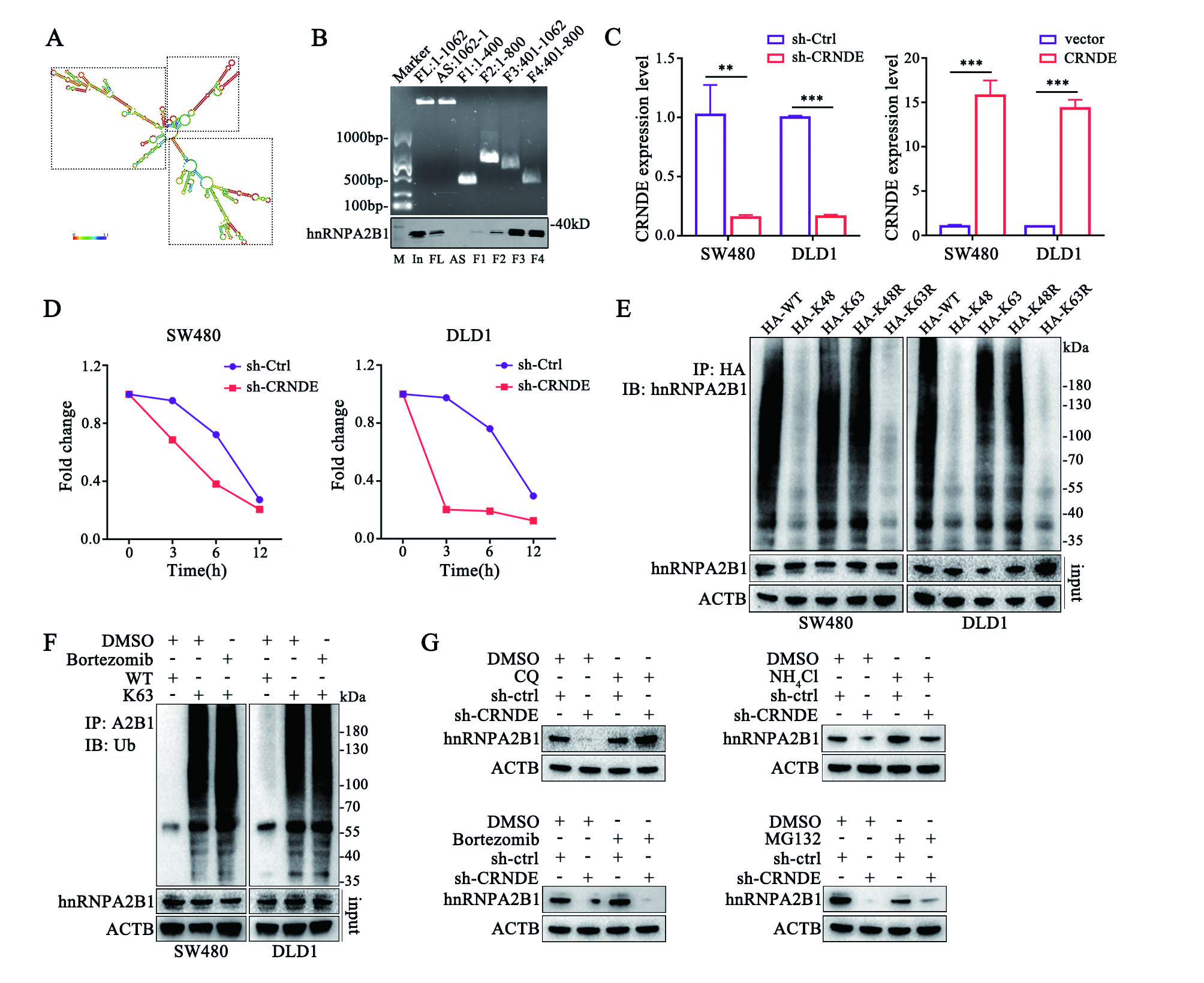


**Figure S4** CRNDE binds and suppresses hnRNPA2B1 protein degradation. **A** Graphic illustration of the CRNDE secondary structure predicted by RNAfold WebServer (http://rna.tbi.univie.ac.at/cgi-bin/RNAWebSuite/RNAfold.cgi). **B** Agarose gel electrophoresis analysis of CRNDE full-length, antisense and truncated fragments (F1, 1-400nt; F2, 1-800nt; F3, 401-1062nt; and F4, 401-800nt) (up panel). The hnRNPA2B1 content in protein samples pulled down by different CRNDE truncates was analyzed by western blot (down panel). **C** Cell lines stably knocking down or overexpressing CRNDE were constructed in SW480 and DLD1 and the efficiency was verified by qPCR. **D** CRNDE-silencing CRC cells were treated with CHX for the indicated times. Densitometry analysis of hnRNPA2B1 protein levels with their relative fold changes compared to the protein level of ACTB. **E** The enrichment of hnRNPA2B1 was detected through HA-IP assays using HA-tagged wild-type, K48, K63, K48R and K63R Ubs in sh-CRNDE CRC cells. **F** CRC cells transfected with K63 Ub were treated with 50 nM Bortezomib for 6 h. The ubiquitin level of hnRNPA2B1 was detected by IP assays. **G** hnRNPA2B1 protein levels in CRNDE stable knockdown cell lines were detected after treatment with MG132 (25 μM), Bortezomib (50 nM), chloroquine (50 μM), and NH_4_Cl (20 mM) for 6 h, respectively. A two-tailed Student’s *t-*test and one-way ANOVA were used for statistical analysis, respectively. ***P＜*0.01, ****P*<0.001. Data represent mean±SD.


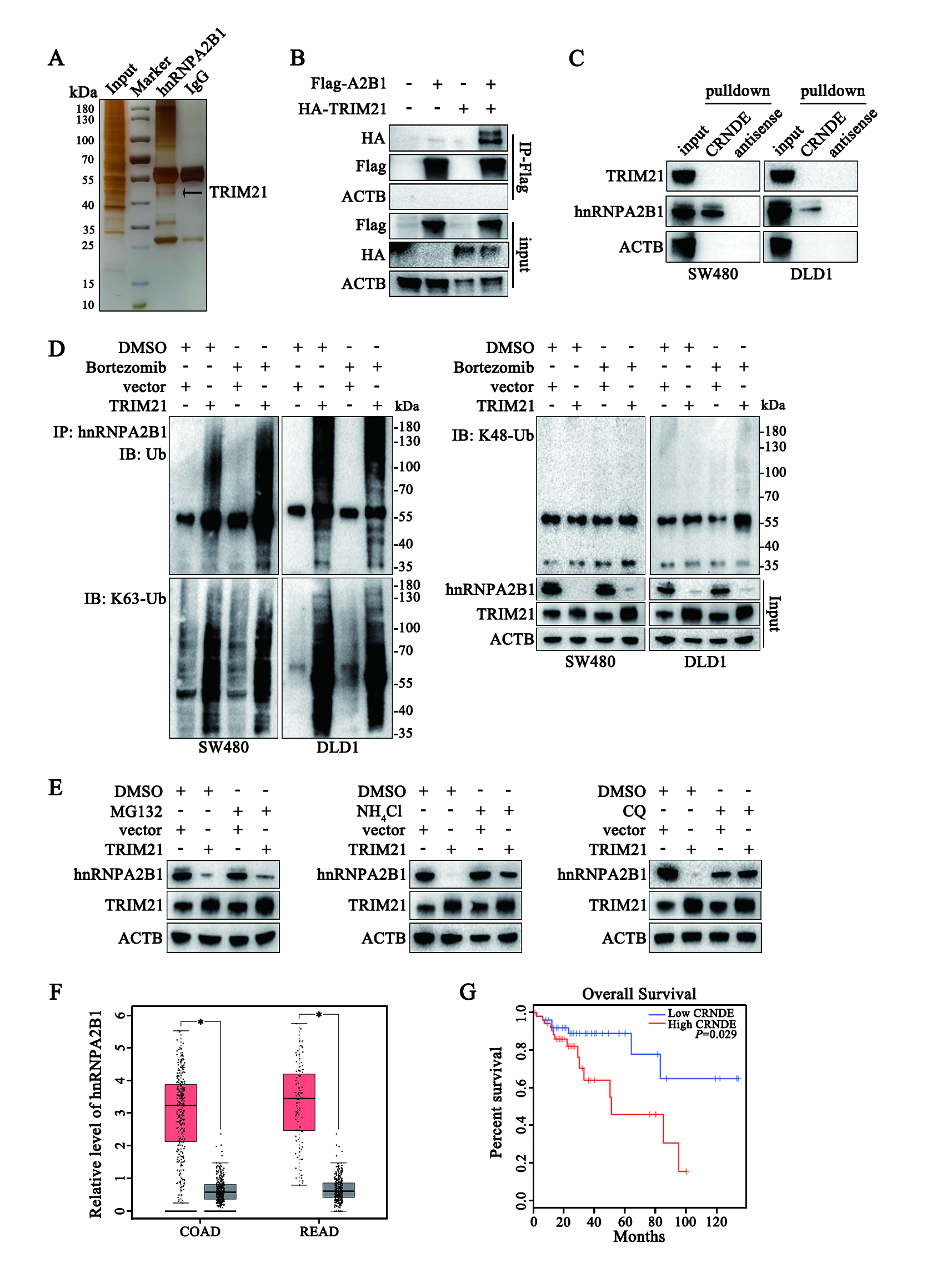


**Figure S5** TRIM21-mediated K63 ubiquitination is involved in the regulation of hnRNPA2B1 expression by CRNDE. **A** The silver-stained image revealed the protein precipitated with hnRNPA2B1. The band was shown by an arrow corresponding to the specific hnRNPA2B1-interacting protein, which was subsequently analyzed and identified by mass spectrometry. **B** The flag-tagged hnRNPA2B1 and HA-tagged TRIM21 overexpression plasmid were co-transfected into 293T cells, followed by in vitro IP assays using FLAG beads. Western blot was applied to identify the binding relationship between exogenous hnRNPA2B1 and TRIM21 by detecting HA. **C** Western blot of TRIM21, hnRNPA2B1 and ACTB in the complexes from CRNDE-pulldown assays. **D** Detection of ubiquitin levels of hnRNPA2B1 upon TRIM21 overexpression with or without Bortezomib (50 nM) treatment using K48/K63 linkage-specific antibodies. **E** MG132 (25 μM), chloroquine (50 μM) and NH_4_Cl (20 mM) were co-incubated with TRIM21 overexpressing cell lines to investigate the protein changes of hnRNPA2B1. **F** Expression of CRNDE in CRC based on the TCGA database of the GEPIA platform. **G** Overall survival analysis of CRNDE in CRC according to the TCGA database. A two-tailed Student’s *t-*test and one-way ANOVA were used for statistical analysis, respectively. **P＜*0.05. Data represent mean±SD.


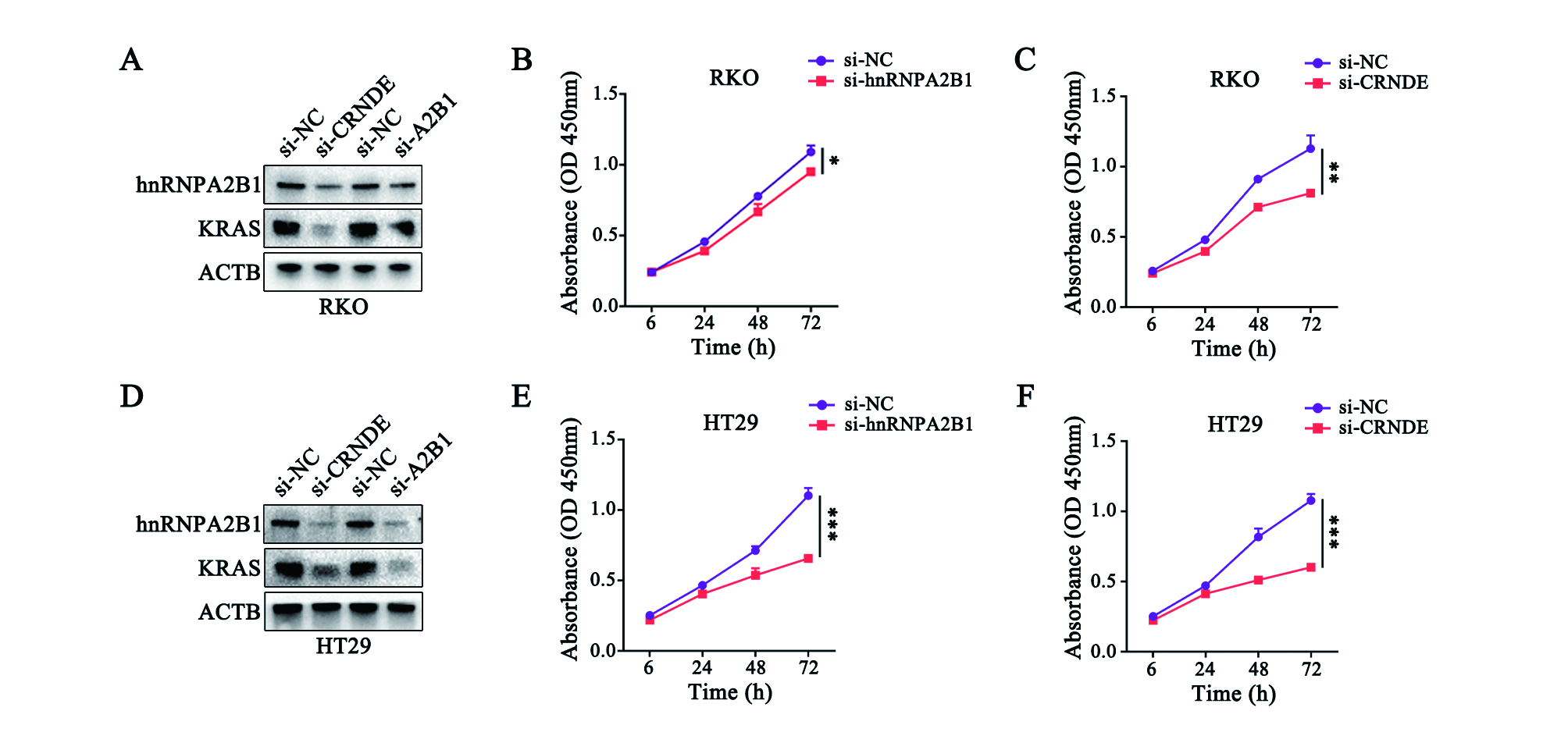


**Figure S6** The role of hnRNPA2B1 in KRAS wild-type CRC cells. **A, D** CRNDE and hnRNPA2B1 were knocked down in non-KRAS mutant CRC cell RKO (A) and HT29 (D), respectively, and the protein changes of hnRNPA2B1 and KRAS were detected by western blot. **B-F** CCK8 assays examined the effect of silencing hnRNPA2B1 (B, E) or CRNDE (C, F) on cell proliferation activity in KRAS wild-type cells RKO (B, C) and HT29 (E, F), respectively. n=3 independent biological replicates. One-way ANOVA was used for statistical analysis. **P＜*0.05, ***P＜*0.01, ****P*<0.001. Data represent mean±SD.


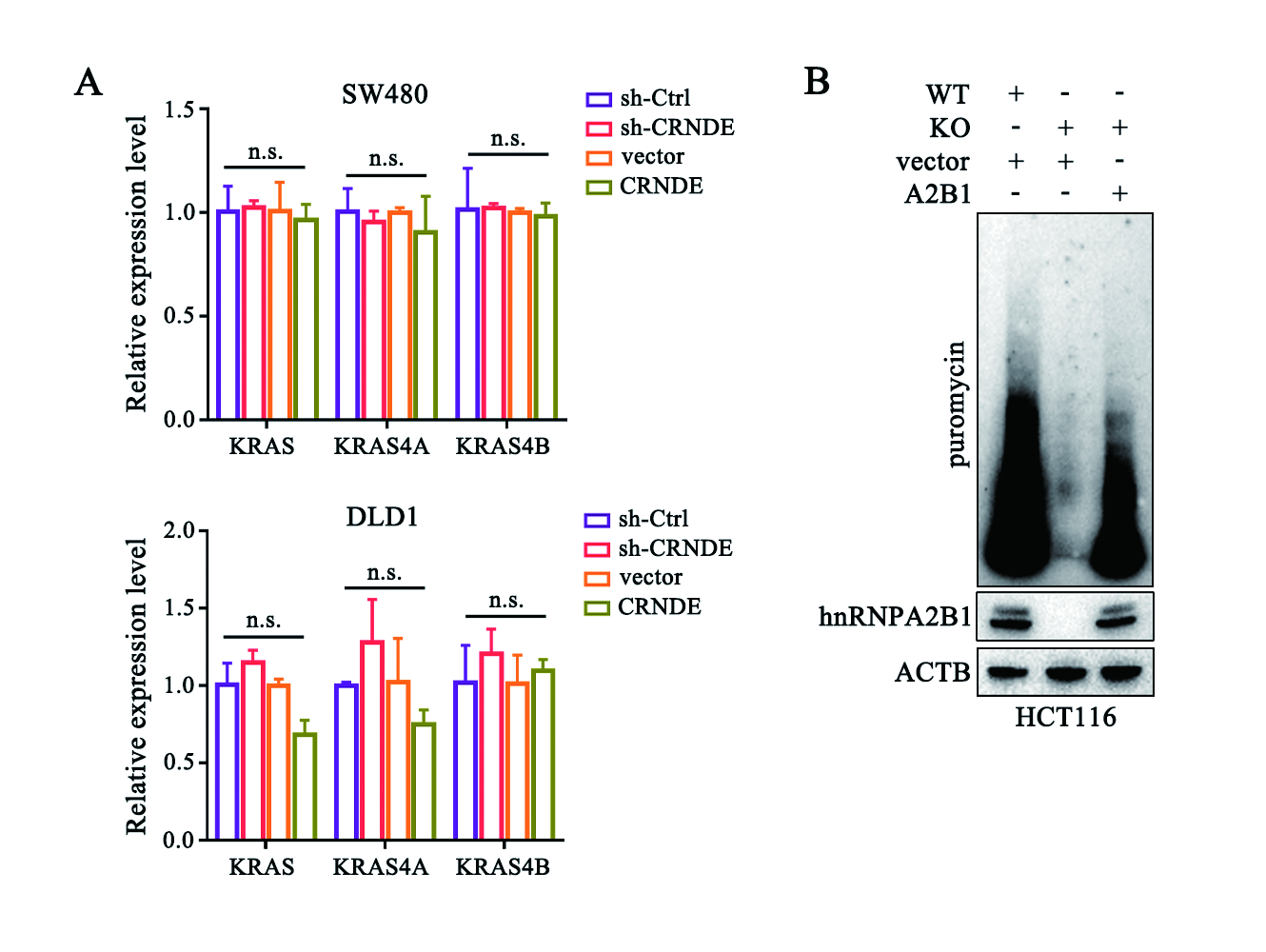


**Figure S7** Alternative splicing of KRAS is not affected by CRNDE. **A** qPCR detection of KRAS and its splice variants KRAS4A and KRAS4B in SW480 (left panel) and DLD1 (right panel) with stable CRNDE knockdown or overexpression. **B** SUnSET experiments were performed in the HCT116 KO cell lines with exogenously hnRNPA2B1 overexpression. A two-tailed unpaired Student’s *t-*test was used for statistical analysis. n.s., not significantly. Data represent mean±SD.

**Supplementary tables**

**Table S1** Correlation between hnRNPA2B1 expression and clinicopathologic features of CRC specimens

| Features | N of | hnRNPA2B1 | |  |
| --- | --- | --- | --- | --- |
|  | cases | High | Low | *P-*value |
| Total | 24 | 18 | 6 |  |
| Age (year) |  |  |  |  |
| ≤60 | 10 | 7 | 3 | 0.665 |
| >60 | 14 | 11 | 3 |  |
| Gender |  |  |  |  |
| Male | 13 | 9 | 4 | 0.649 |
| Female | 11 | 9 | 2 |  |
| Differentiation |  |  |  |  |
| Well and moderately | 11 | 7 | 4 | 0.357 |
| Poorly | 13 | 11 | 2 |  |
| Location |  |  |  |  |
| Colon | 13 | 10 | 3 | >0.999 |
| Rectum | 11 | 8 | 3 |  |
| Tumor size (cm) |  |  |  |  |
| ≤5 | 12 | 9 | 3 | >0.999 |
| >5 | 12 | 9 | 3 |  |
| Depth of invasion |  |  |  |  |
| T1 | 1 | 1 | 0 | ***0.015**** |
| T2 | 3 | 0 | 3 |  |
| T3 | 12 | 10 | 2 |  |
| T4 | 8 | 7 | 1 |  |
| Lymph node metastasis |  |  |  |  |
| Present | 14 | 12 | 2 | 0.192 |
| Absent | 10 | 6 | 4 |  |
| Distant metastasis |  |  |  |  |
| Present | 4 | 4 | 0 | 0.539 |
| Absent | 20 | 14 | 6 |  |
| AJCC stage |  |  |  |  |
| I | 4 | 1 | 3 | 0.071 |
| II | 6 | 5 | 1 |  |
| III | 10 | 8 | 2 |  |
| IV | 4 | 4 | 0 |  |

**Table S2** Mass spectrometry results of hnRNPA2B1 immunoprecipitated protein mixture

| **Number** | **Accession** | **Gene ID** | **Unique Peptides** |
| --- | --- | --- | --- |
| 1 | Q9BQA1 | WDR77 | 11 |
| 2 | O75688 | PPM1B | 7 |
| 3 | P68104 | EEF1A1 | 6 |
| 4 | P22626 | HNRNPA2B1 | 6 |
| 5 | P19474 | TRIM21 | 5 |
| 6 | Q06830 | PRDX1 | 4 |
| 7 | P68363 | TUBA1B | 3 |
| 8 | P31943 | HNRNPH1 | 3 |
| 9 | O14744 | PRMT5 | 3 |
| 10 | Q8WUA2 | PPIL4 | 2 |
| 11 | P00338 | LDHA | 2 |
| 12 | P07437 | TUBB | 2 |
| 13 | Q96I25 | RBM17 | 2 |
| 14 | A6NMY6 | ANXA2P2 | 2 |

**Table S3** Genes and primers for qRT-PCR

| **Genes** | **Sequences(from 5’ to 3’)** |
| --- | --- |
| GAPDH | F: GGATTTGGTCGTATTGGGCG |
|  | R: ATCGCCCCACTTGATTTTGG |
| 18SRNA | F: CGAACGTCTGCCCTATCAACTT |
|  | R: ACCCGTGGTCACCATGGTA |
| CRNDE | F: TTCAGCCGTTGGTCTTTG |
|  | R: CTGCGTGACAACTGAGGATT |
| hnRNPA2B1 | F: TATGGCAGTGGACGTGGATT |
|  | R: TCATAACCACCTCCGTAGCC |
| TRIM21 | F: ATGATGTGGGAGGAGGTCAC |
|  | R: GTTGGCTAGCTGTCGATTGG |
| KRAS | F: ACACAAAACAGGCTCAGGAC |
|  | R: TCACACAGCCAGGAGTCTTT |
| KRAS 4A | F: TGTGATTTGCCTTCTAGAACAGTAGAC |
|  | R: CTCACCAATGTATAAAAAGCATCCTC |
| KRAS 4B | F: TGAGGACTGGGGAGGGCTTT |
|  | R: AGGCATCATCAACACCCTGTCT |

**Table S4** Oligonucleotide sequences for siRNA

| **Genes** | **Sequences** |
| --- | --- |
| NC siRNA | 5’ UUCUCCGAACGUGUCACGUTT 3’ |
|  | 5’ ACGUGACACGUUCGGAGAATT 3’ |
| hnRNPA2B1 siRNA | 5’ GAGAUUACUUUGAGGAAUA 3’ |
| CRNDE siRNA | 5’ TATGGAAGCATCACACTTAACACCT 3’ |
| TRIM21 siRNA | 5’ GUGAACAACCUUAAAGAAA 3’ |
|  | 5’ UUUCUUUAAGGUUGUUCAC 3’ |
| KRAS siRNA | 5’ CGAAUAUGAUCCAACAAUA 3’ |
|  | 5’ UAUUGUUGGAUCAUAUUCG 3’ |
| USP33 siRNA | 5’ CAGCAGAGCUUCAGAAUAU 3’ |
|  | 5’ AUAUUCUGAAGCUCUGCUG 3’ |
