# Supplementary material for: CRNDE mediated hnRNPA2B1 stability facilitates nuclear export and translation of KRAS in colorectal cancer
Source: Cell Death Dis. 2023 Sep 16;14(9):611. doi: 10.1038/s41419-023-06137-9 (PMC10505224; doi:10.1038/s41419-023-06137-9)

Figure 2D

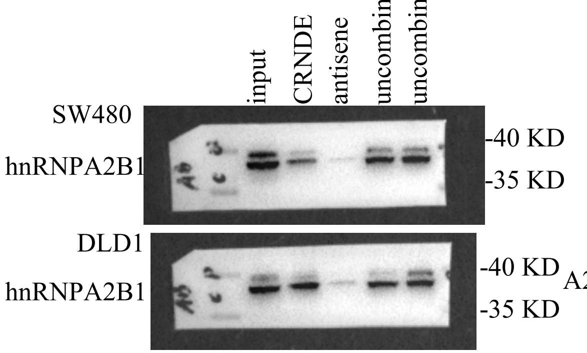

Figure 2F

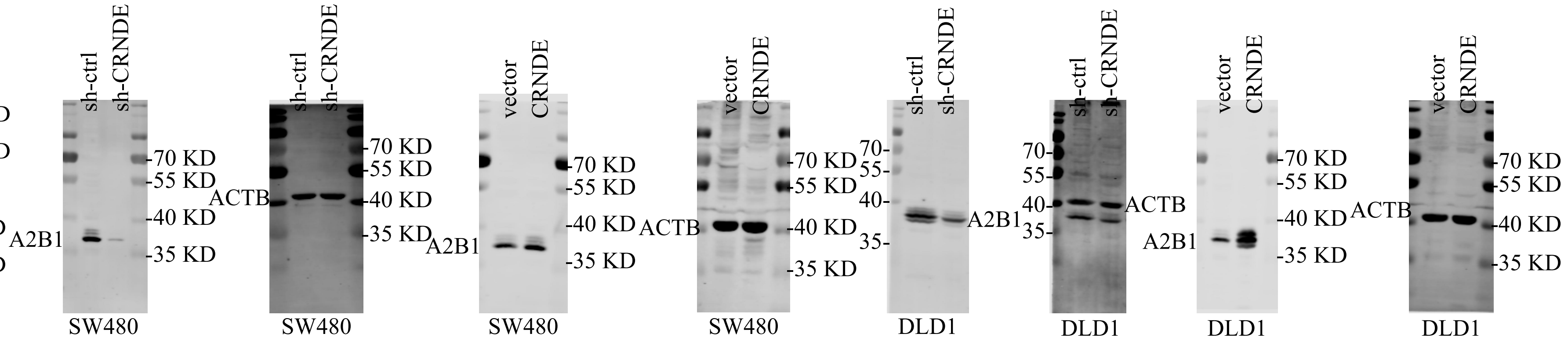

Figure 2I

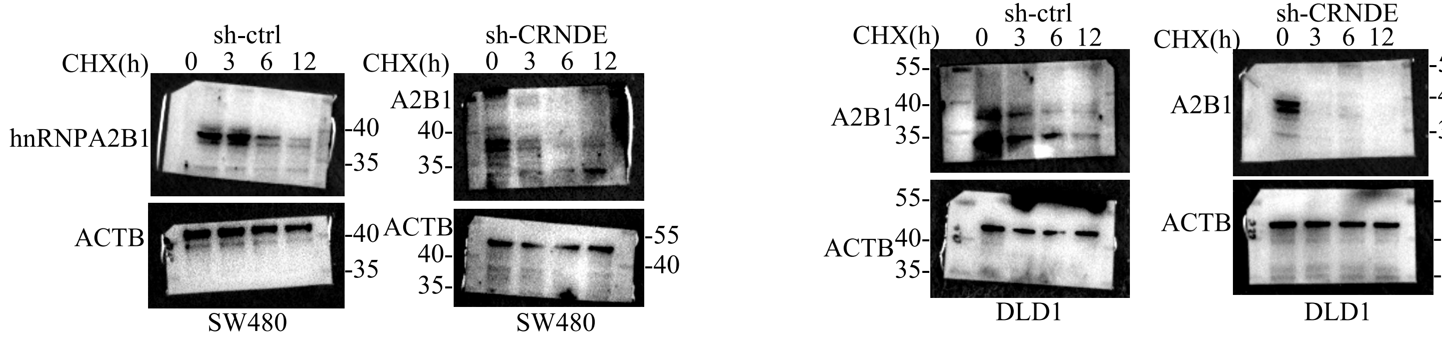

Figure 2J

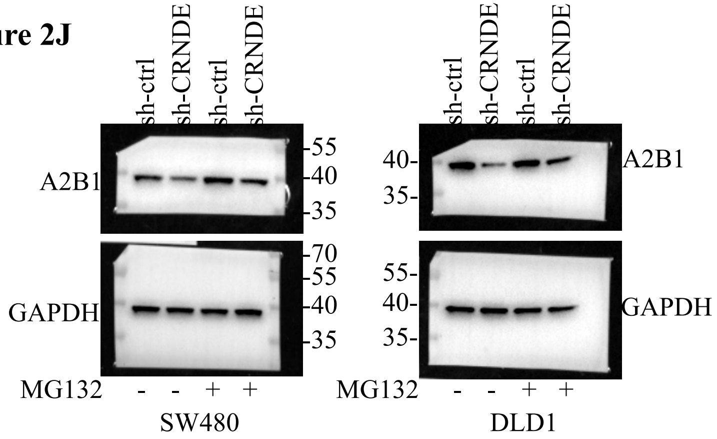

Figure 2K

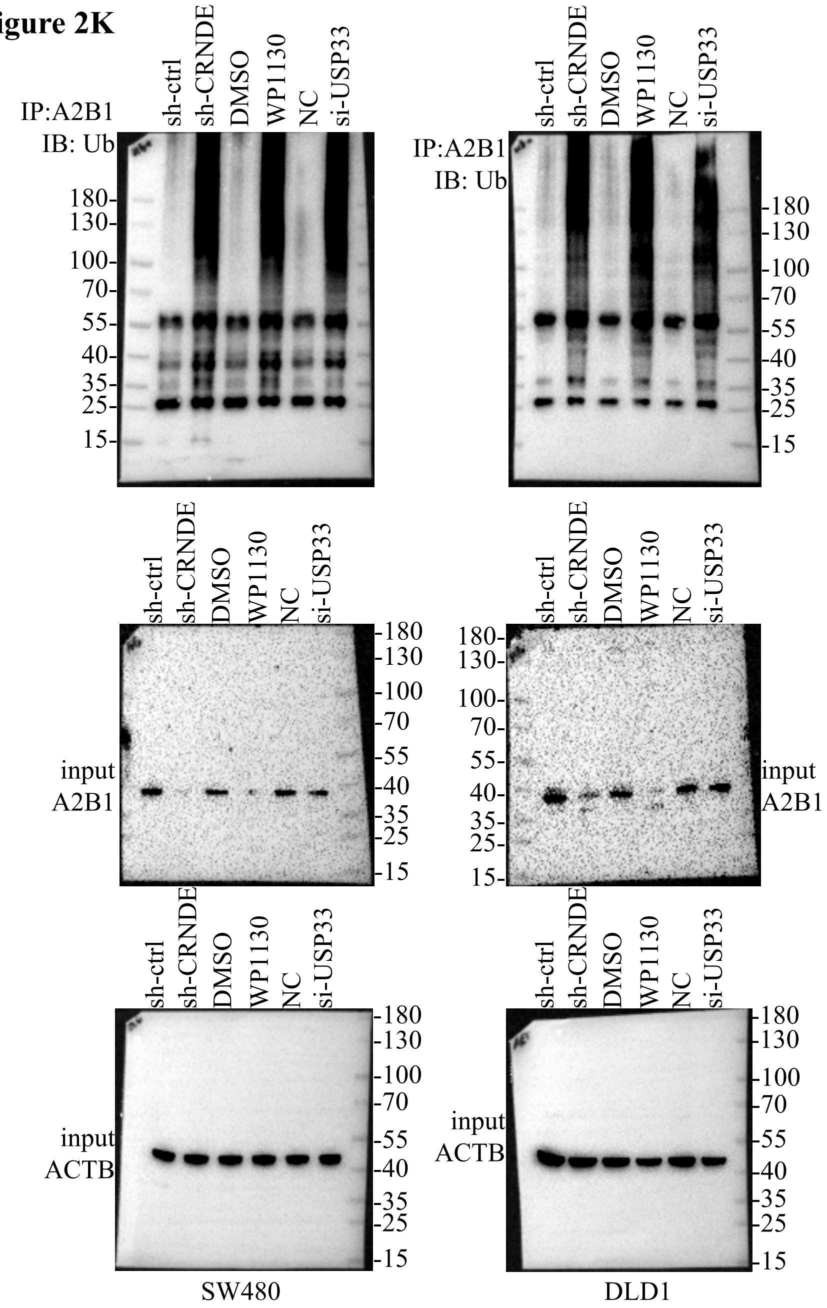

Figure 2L

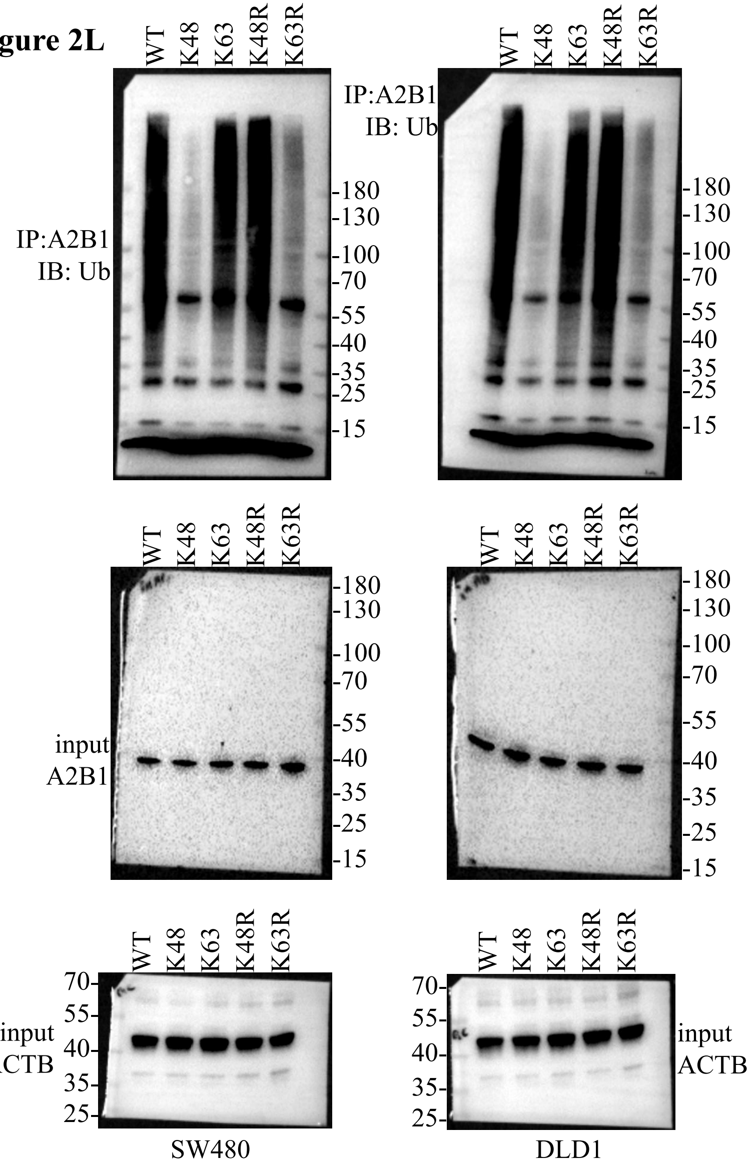

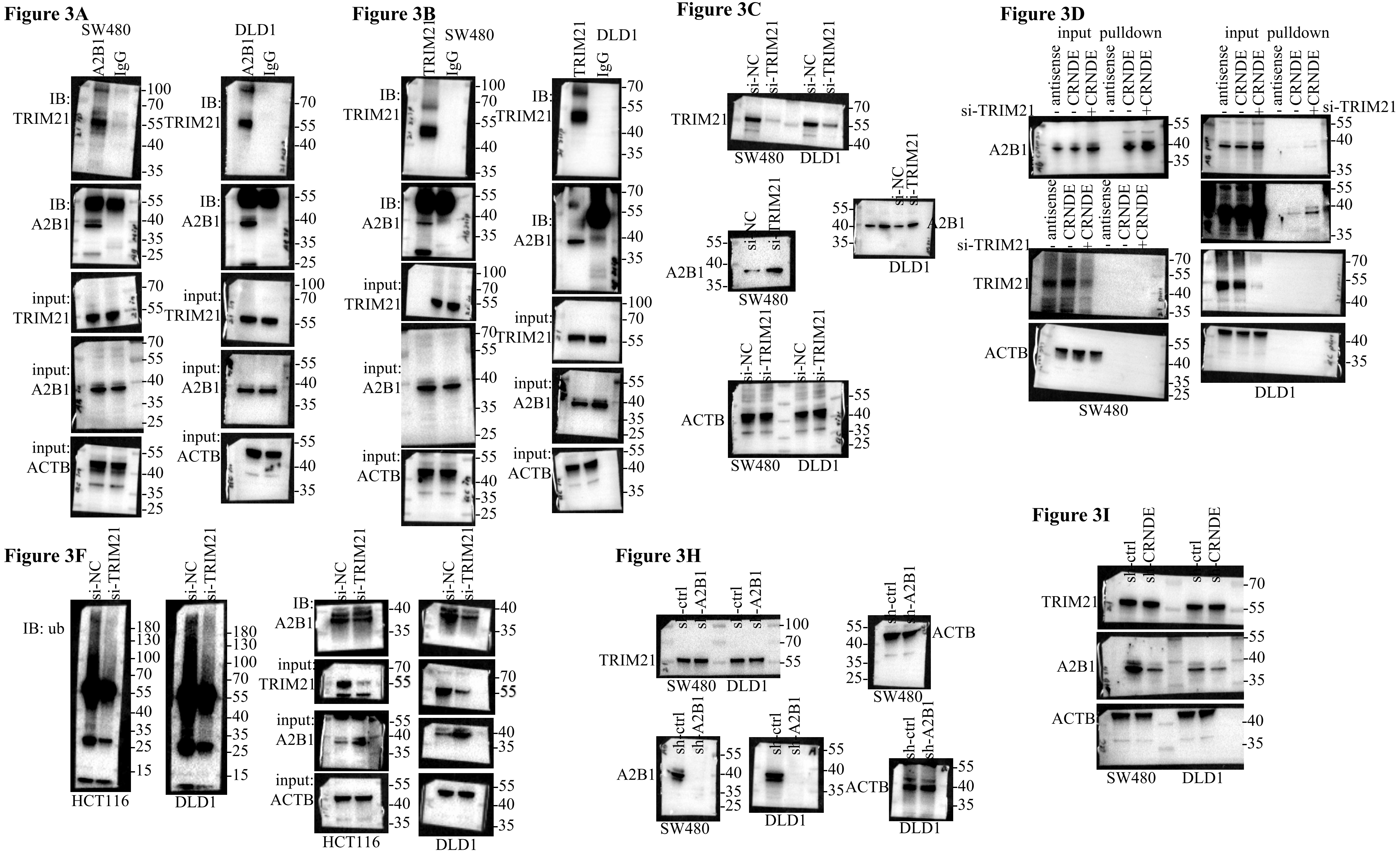

Figure 3A
SW480
DLD1
A2B1
IgG
IB: TRIM21
IB: A2B1
input: TRIM21
input: A2B1
input: ACTB
100
70
55
40
35
25
Figure 3B
SW480
DLD1
TRIM21
IgG
IB: TRIM21
IB: A2B1
input: TRIM21
input: A2B1
input: ACTB
Figure 3C
si-NC
si-TRIM21
TRIM21
A2B1
ACTB
SW480
DLD1
Figure 3D
input
pulldown
antisense
CRNDE
si-TRIM21
A2B1
TRIM21
ACTB
SW480
DLD1
Figure 3F
si-NC
si-TRIM21
IB: ub
180
130
15
HCT116
DLD1
IB: A2B1
input: TRIM21
input: A2B1
input: ACTB
Figure 3H
sh-ctrl
sh-A2B1
TRIM21
A2B1
ACTB
SW480
DLD1
Figure 3I
sh-ctrl
sh-CRNDE
TRIM21
A2B1
ACTB
SW480
DLD1

Figure 3J

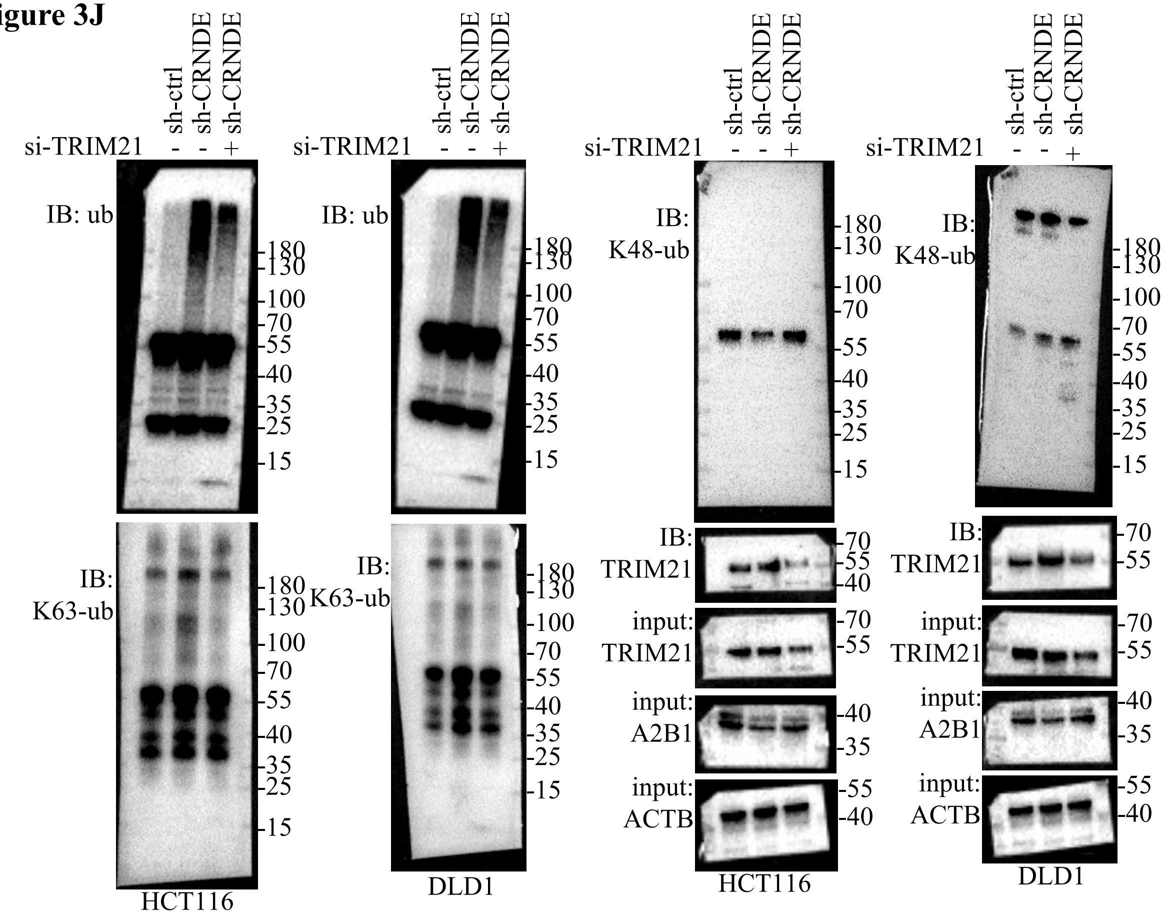

Figure 3K

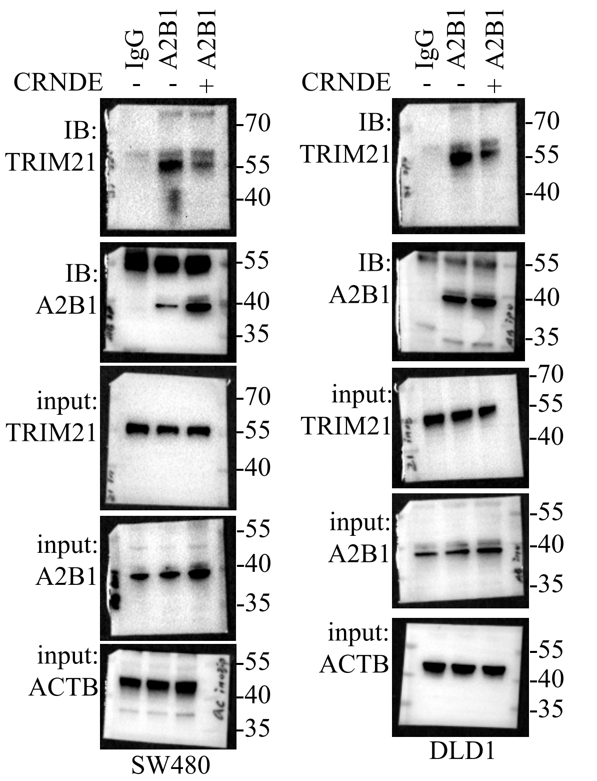

Figure 5A-B

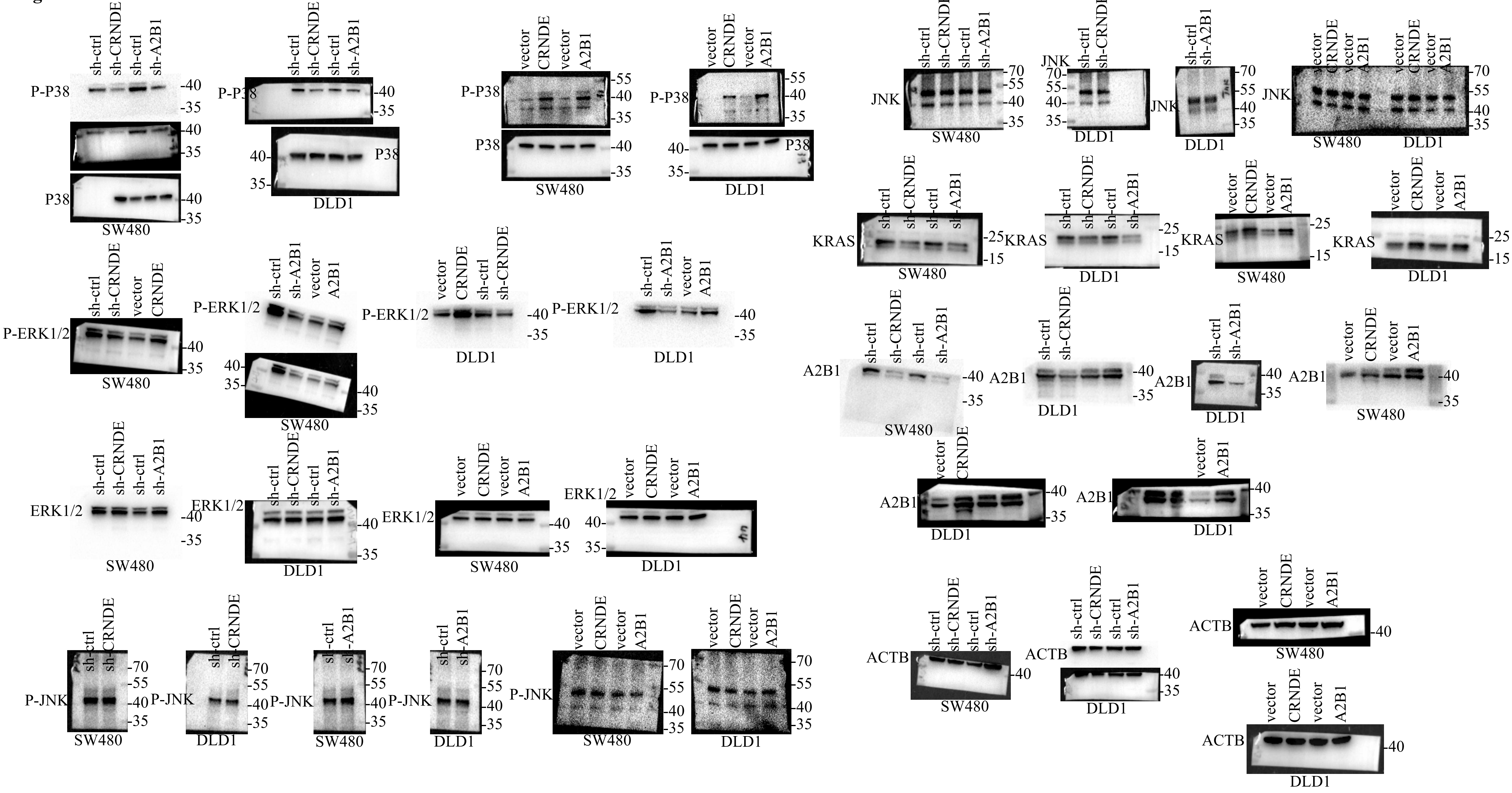
sh-ctrl
sh-CRNDE
sh-ctrl
sh-A2B1
vector
CRNDE
vector
A2B1
P-P38
P38
P-ERK1/2
ERK1/2
P-JNK
JNK
KRAS
A2B1
ACTB
SW480
DLD1
70
55
40
35
25
15

Figure 5E

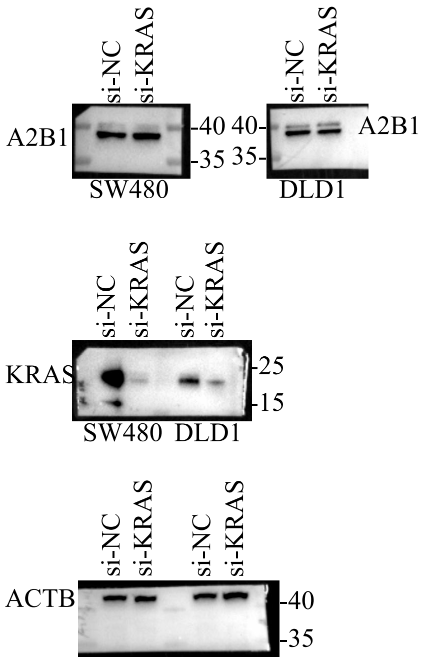
si-NC
si-KRAS
A2B1
-40
-35
SW480
40-
35-
DLD1
KRAS
-25
-15
SW480
DLD1
ACTB

Figure 6A

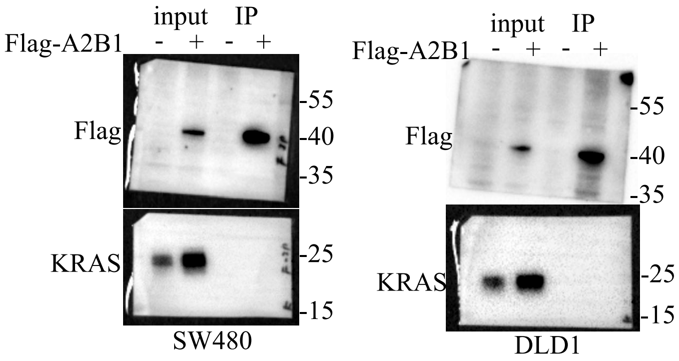

Figure 6B

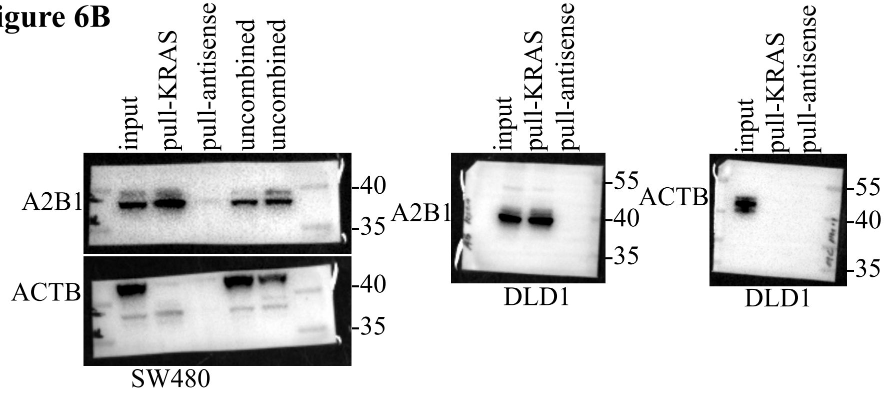

Figure 6D

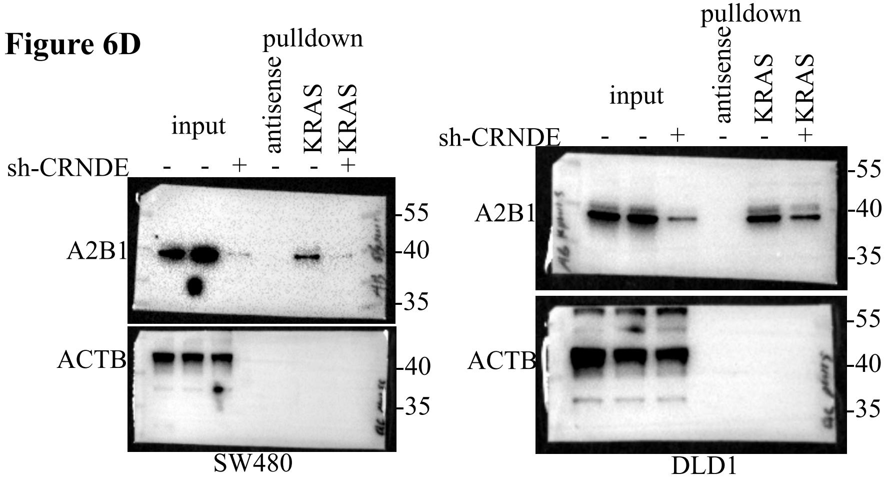

Figure 6E

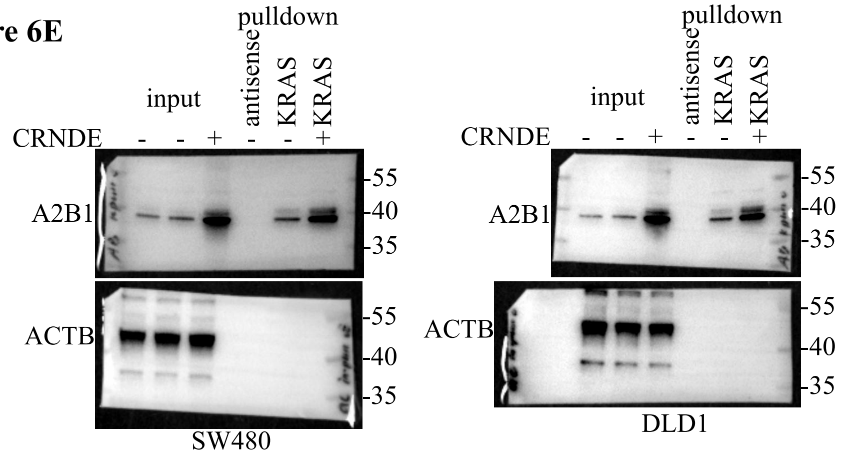

Figure 6J

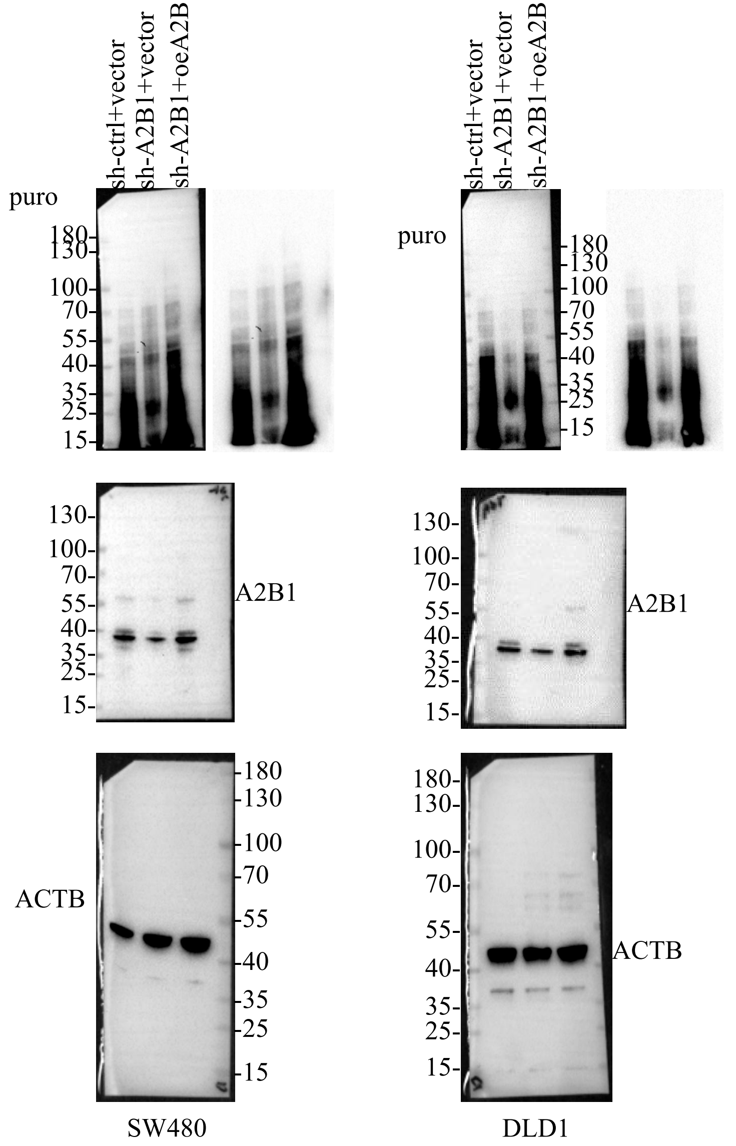

Figure 6K

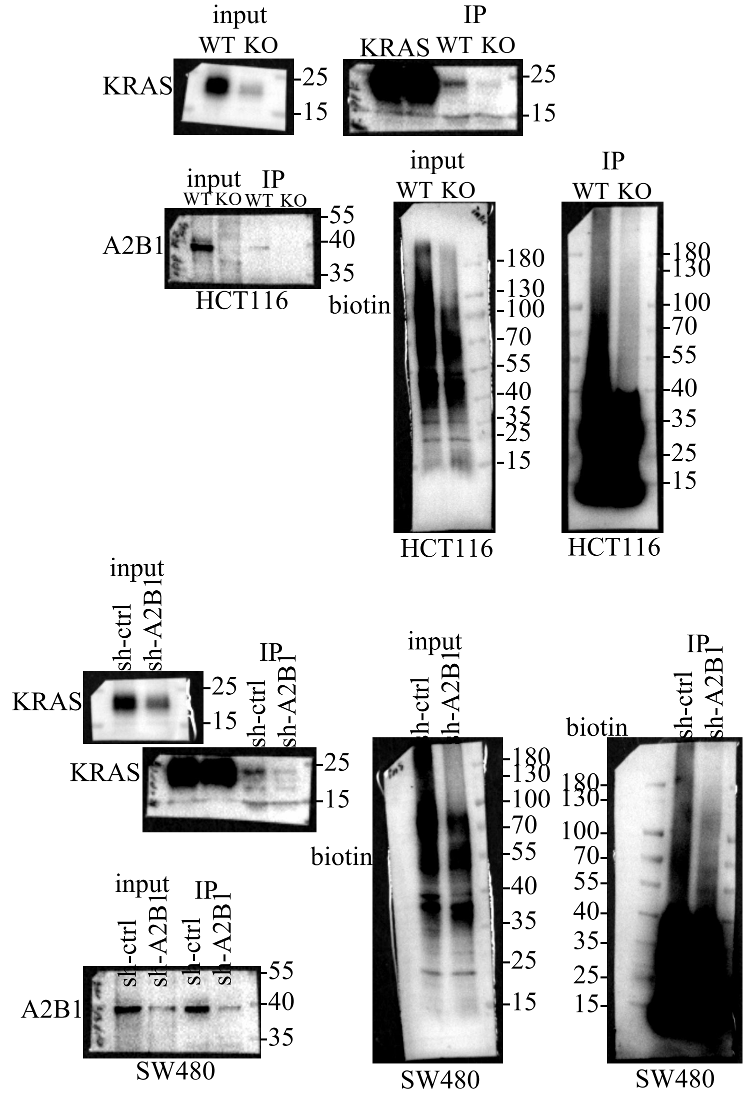

Figure 6M

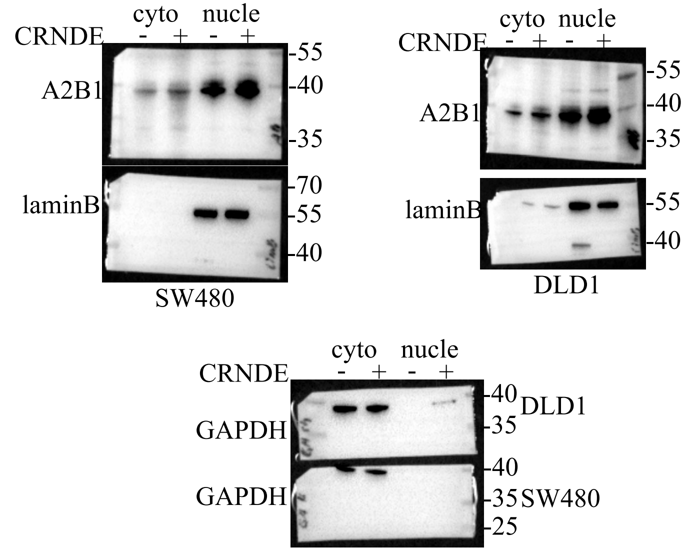

Figure S1B

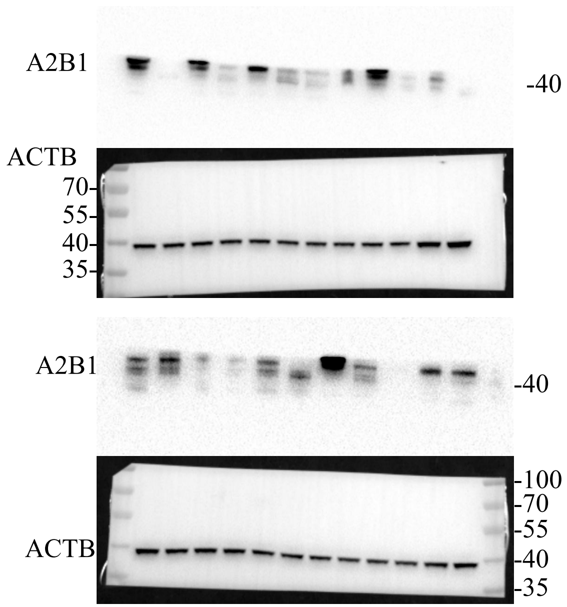

Figure S1D

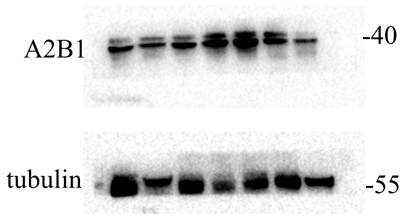

Figure S2A

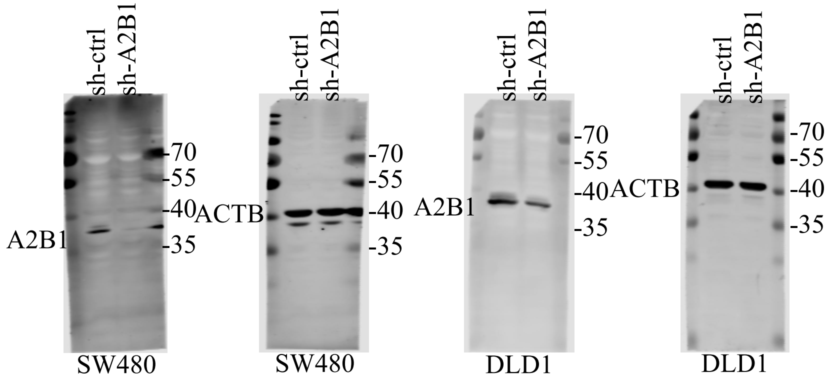

Figure S2H

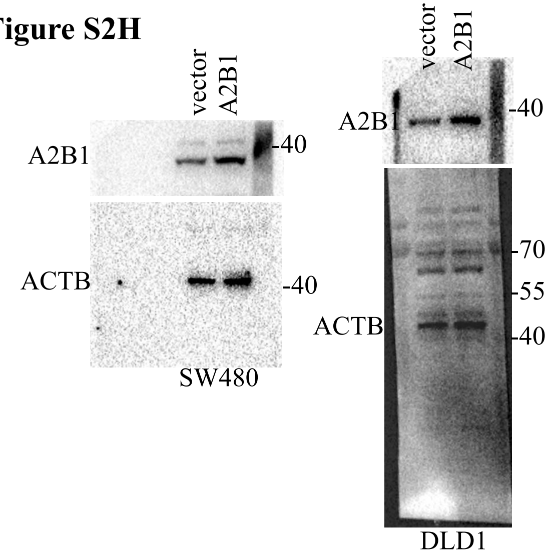

Figure S3A

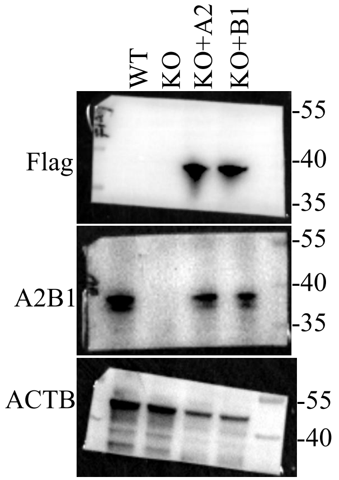

Figure S4B

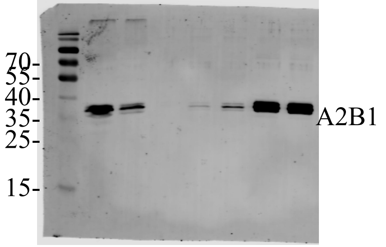

Figure S4E

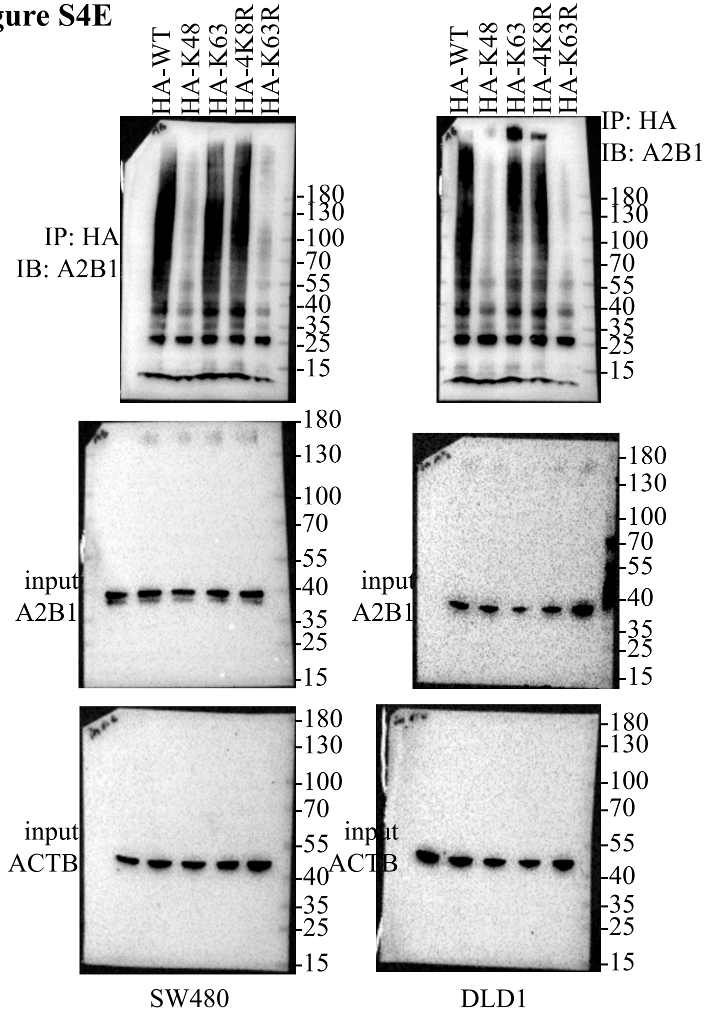

Figure S4F

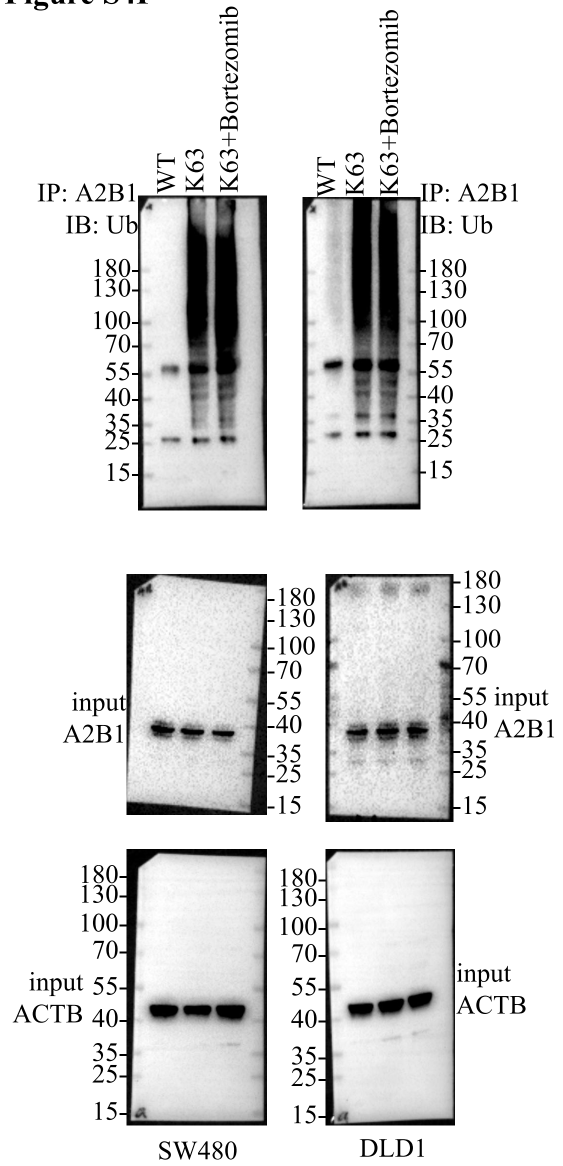

Figure S4G

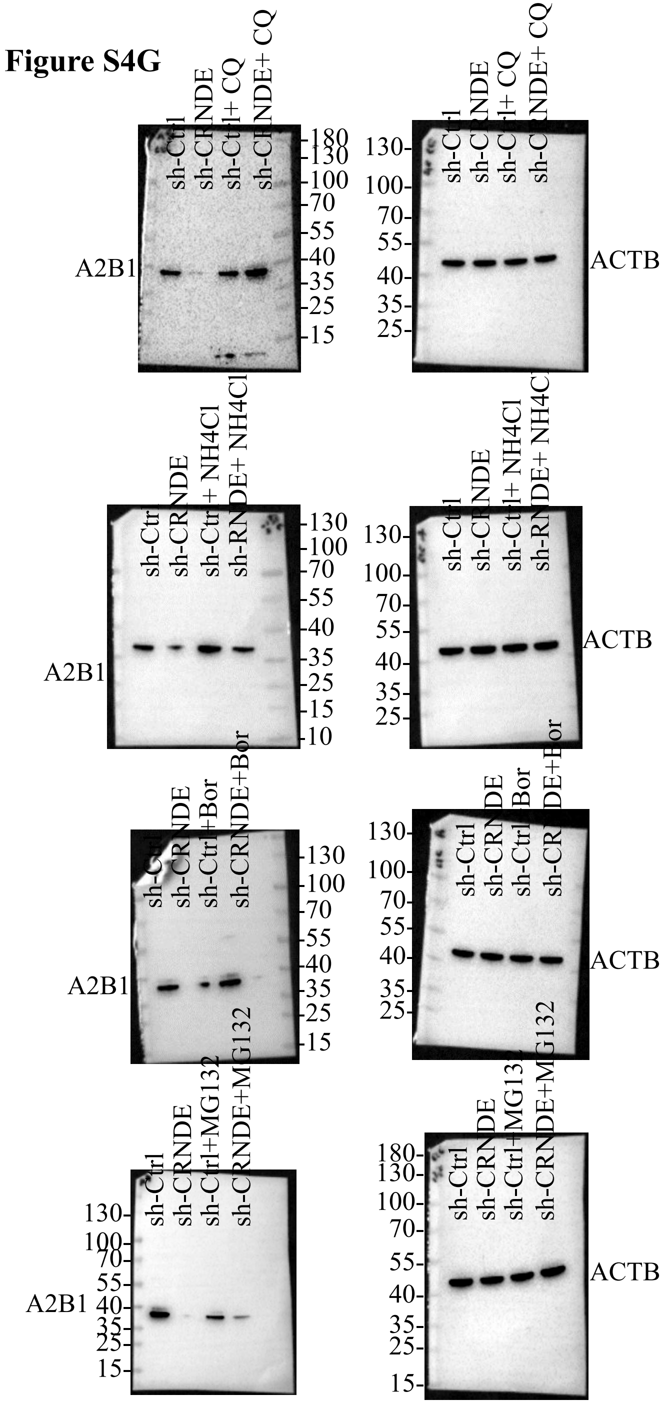

Figure S5B

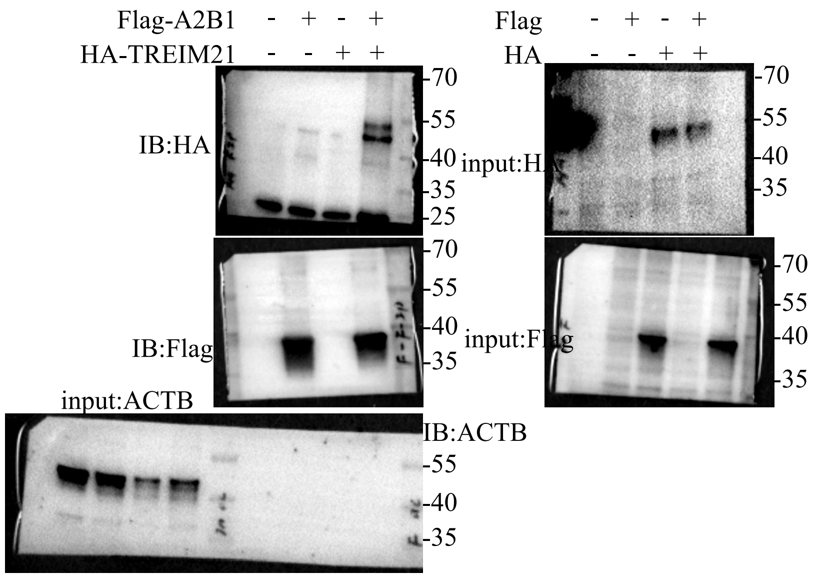

Figure S5C

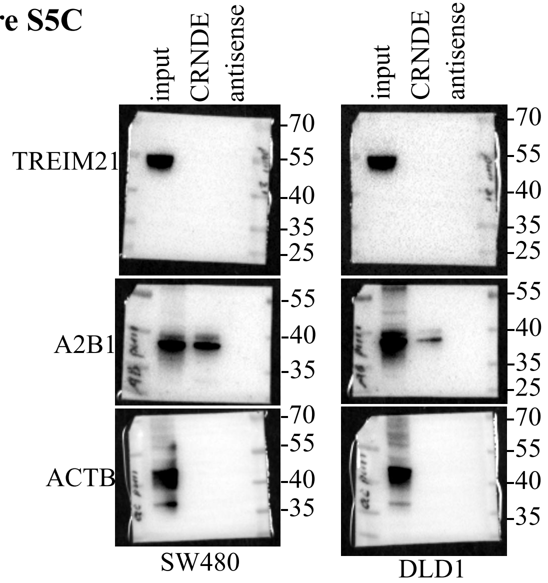

Figure S5D

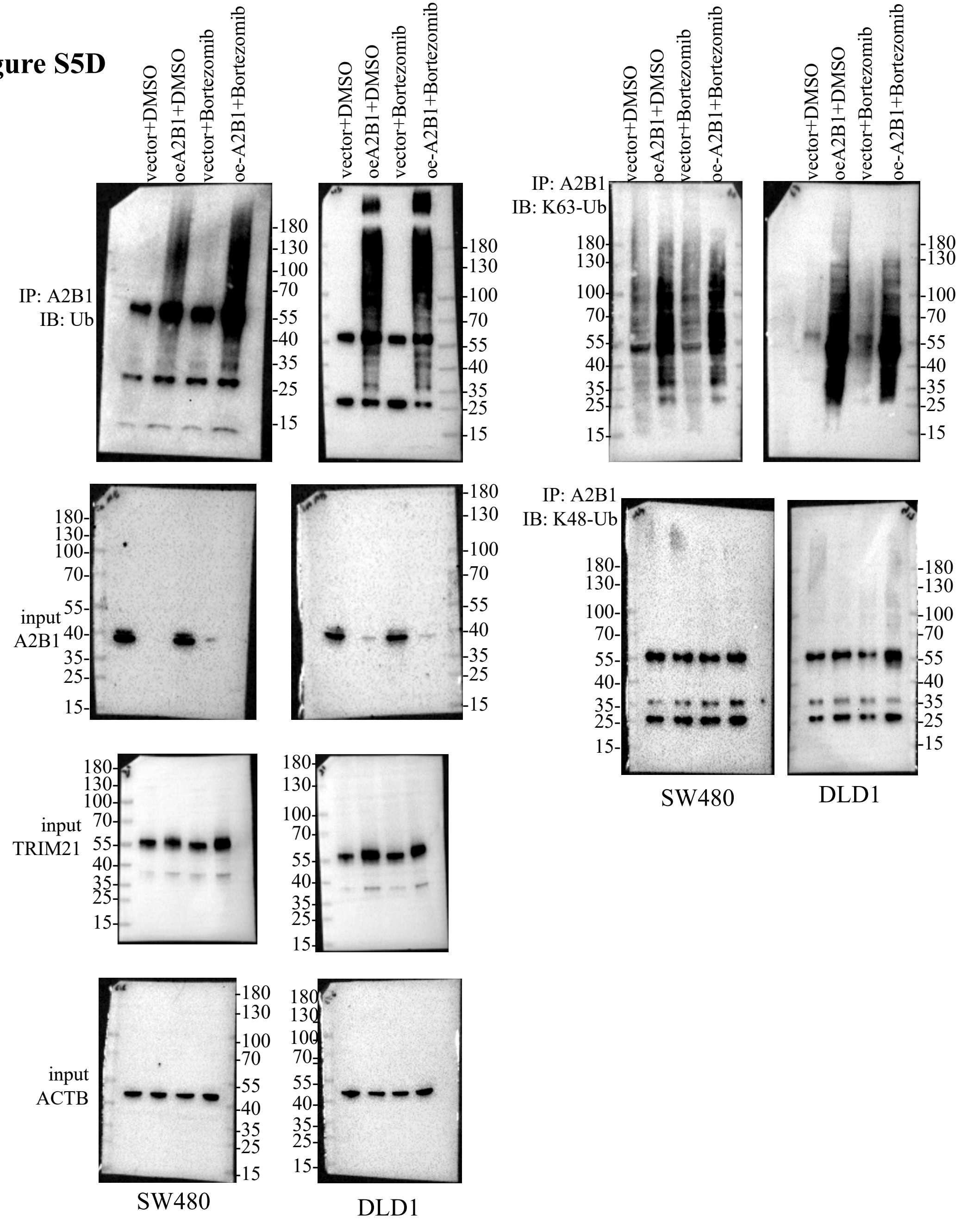

Figure S5E

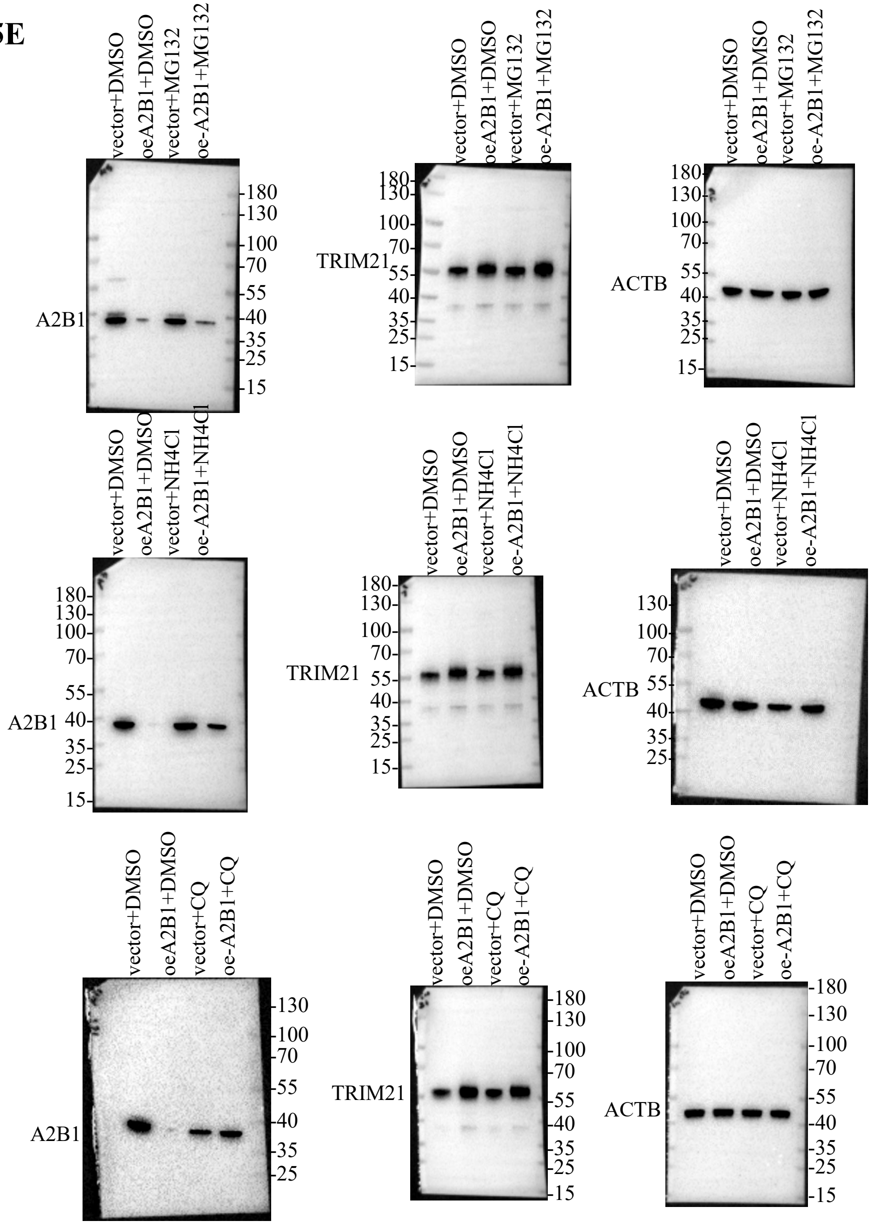
vector+DMSO
oeA2B1+DMSO
vector+MG132
oe-A2B1+MG132
A2B1
TRIM21
ACTB
vector+NH4Cl
oe-A2B1+NH4Cl
vector+CQ
oe-A2B1+CQ
180
130
100
70
55
40
35
25
15

Figure S6A

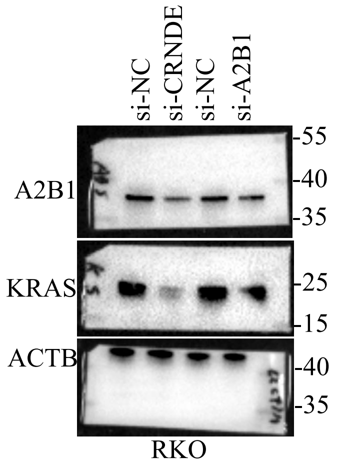

Figure S6D

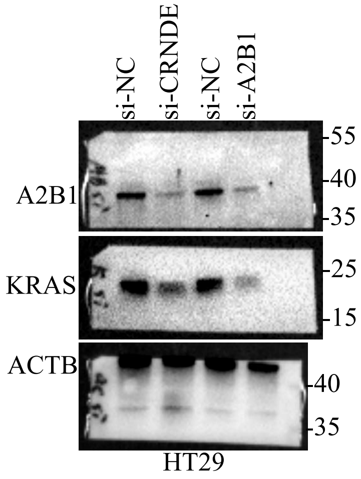

Figure S7B

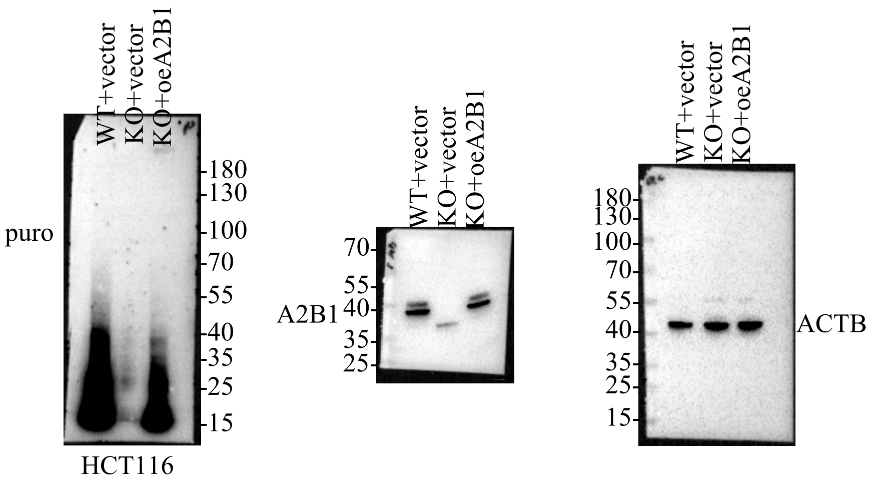

Supplement: Supplementary file 2 — Original western blots [file 41419_2023_6137_MOESM2_ESM.pdf]
